# Sequence and biochemical analysis of vaccinia virus A32 protein: Implications for *in vitro* stability and coiled-coil motif mediated regulation of the DNA-dependent ATPase activity

**DOI:** 10.1371/journal.pone.0316818

**Published:** 2025-01-06

**Authors:** Uma Ramakrishnan, Tanvi Aggarwal, Kiran Kondabagil

**Affiliations:** Department of Biosciences and Bioengineering, Indian Institute of Technology Bombay, Mumbai, India; East China Normal University School of Life Sciences, CHINA

## Abstract

Nucleocytoplasmic large DNA viruses (NCLDVs) have massive genome and particle sizes compared to other known viruses. NCLDVs, including poxviruses, encode ATPases of the FtsK/HerA superfamily to facilitate genome encapsidation. However, their biochemical and structural characteristics are yet to be discerned. In this study, we demonstrate that the viral ATPases are significantly shorter than their bacterial homologs, representing the minimal ATPase core of the FtsK/HerA superfamily. We analysed the sequence and secondary structural features of the vaccinia virus A32 protein and determined their roles in the protein’s ATPase activity. We sought to purify A32 by various techniques and noted that recombinant A32 expressed in *E*. *coli* is highly insoluble and unstable in solution. N-terminal fusion with the thioredoxin solubility tag could alleviate this issue to some extent, but subsequent tag cleavage results in increased susceptibility to precipitation and degradation. We have also predicted a highly conserved coiled-coil motif (CCM) towards the C-terminus of vaccinia virus A32. ATPase activity of A32 is known to increase in the presence of DNA. Comparative analysis of the wildtype protein versus its CCM mutants suggests that this DNA dependence of A32’s ATPase activity is likely regulated by the CCM. We demonstrate that oligomerization of A32, mediated by the CCM, is required for its DNA-binding but is not dependent on ATP- or DNA-binding. Our findings suggest a key role of the CCM, and thus, higher-order structure formation in the regulated ATPase activity of A32, providing new opportunities for further detailed characterization of the poxvirus genome packaging process.

## Introduction

Genome packaging is an indispensable step of virus assembly. Double-stranded DNA/RNA viruses with genomes larger than 20 kb generally utilize NTPase motor proteins to translocate their genomes into pre-formed capsids [1]. Interestingly, three disparate groups of viruses, all requiring translocation of DNA through a lipid membrane for its encapsidation, encode putative genome packaging ATPases belonging to the FtsK/HerA superfamily of P-loop NTPases, suggesting that the fundamental principle of energy utilization for the process is highly conserved. These include the *Nucleocytoviricota* phylum (also known as nucleocytoplasmic large dsDNA viruses, NCLDVs), membrane containing dsDNA bacteriophages of the PRD-1 lineage, and filamentous ssDNA phages (*Inoviridae*) [2, 3]. The ATPases typically comprise Walker A and Walker B motifs and a third characteristic motif with a conserved arginine and a polar residue [2]. The conserved arginine likely acts as an arginine finger that binds to the γ-phosphate of ATP in the active site of the neighbouring subunit [4]. The polar residue, mostly glutamine, senses the triphosphate of ATP and activates its hydrolysis [5].

Although different mechanisms for the assembly of filamentous phages [6] and membrane-containing phages [7] have been postulated, the mechanisms of action of the genome packaging machineries in all three groups of viruses are not well understood, probably due to difficulties in obtaining the pure recombinant motor proteins. The genome packaging of NCLDVs is unique in the aspect of the huge genomes that must be packaged. Perhaps, the most extensively studied NCLDV is vaccinia virus, the proto-type virus of the *poxviridae* family [8]. Phylogenetic analysis shows that the putative packaging ATPases of *poxviridae* family, represented by the *A32L* gene product, form a distinct clade within the NCLDVs [9]. Infection using conditional lethal mutant of vaccinia virus encoding an inducible *A32L* gene produced genome deficient, non-infectious virus particles upon repression of A32 protein expression; thus, establishing its necessity in the viral genome packaging [10]. Previous studies with thioredoxin-tagged A32 protein of Goatpox virus [11] and Orf virus [12] have demonstrated their DNA-dependent and divalent metal ion-dependent ATPase activities. However, it is generally preferable for the protein to be produced in its native untagged form for biochemical and structural characterization, to avoid interference by the tag [13]. As per the established nomenclature, we refer to the gene encoding the putative genome packaging ATPase of vaccinia virus as ‘*A32L*’ and the corresponding protein as ‘A32’ [14].

In this study, we show that the viral ATPases correspond to the minimal functional ATPase domains of their bacterial FtsK homologs with some variations. One such variation is a putative coiled-coil motif towards the C-terminus of poxvirus A32 proteins. We report various methods that were attempted to obtain pure recombinant A32 protein of vaccinia virus, including bacterial cytoplasmic expression with hexahistidine affinity tag, cleavable thioredoxin and GST solubility tags, periplasmic expression, and the baculovirus expression system. We observed that A32 is highly insoluble and requires thioredoxin tag fusion for its stabilization. With the thioredoxin-tagged A32, we further show that the coiled-coil motif regulates the DNA-dependence of its ATPase activity, possibly by facilitating protein oligomerization.

## Results and discussion

### Viral putative genome packaging ATPases form a conserved β-sheet core

**Fig 1:**
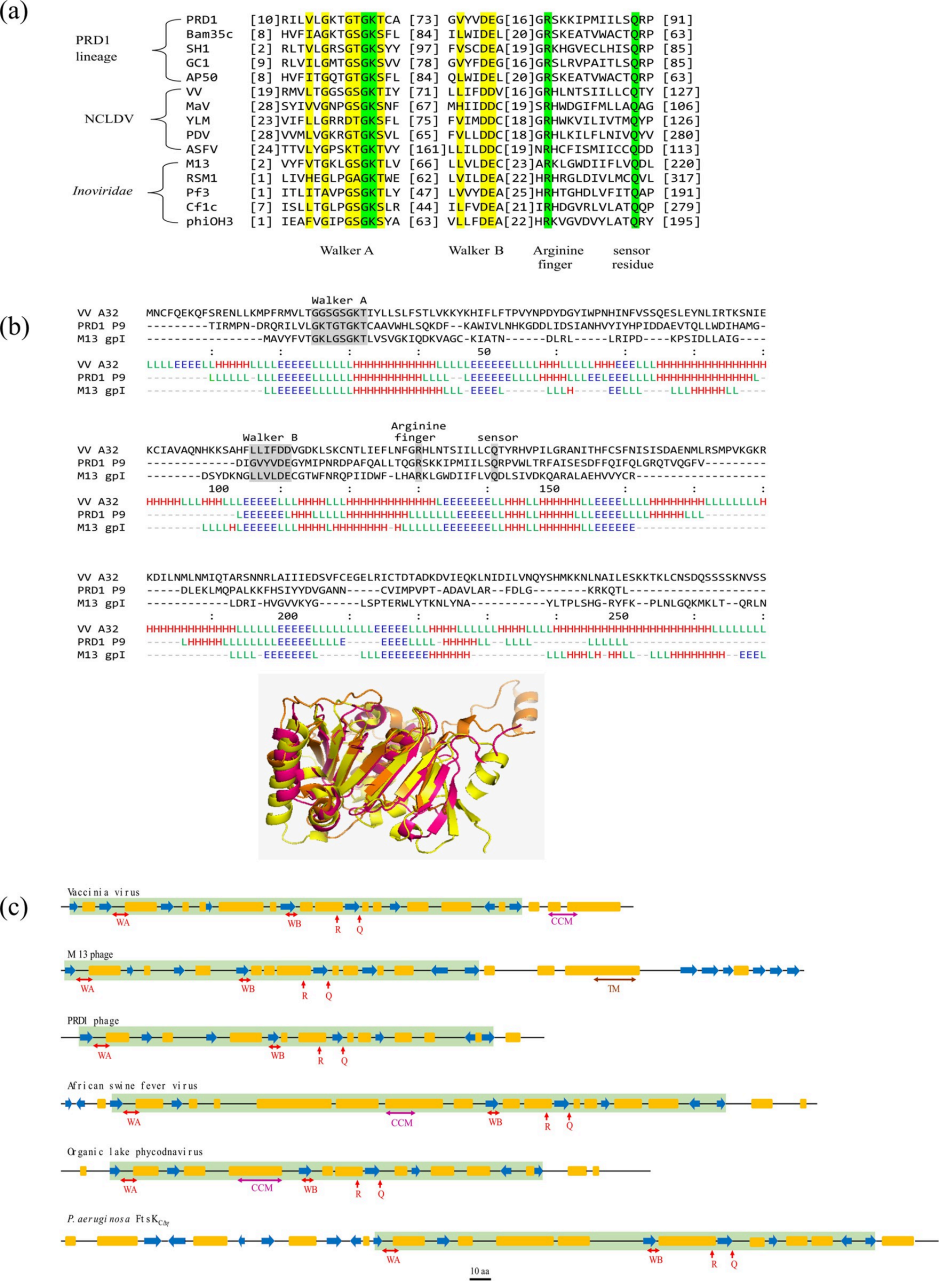
Sequence and structure analysis of viral FtsK-like genome packaging ATPases. (a) Multiple sequence alignment of putative genome packaging ATPases of NCLDVs, PRD1 lineage (membrane-containing dsDNA viruses) and *Inoviridae* family (ssDNA filamentous phages). Green- strictly conserved identical residues; yellow- similar residues (b) Structural alignment of representative ATPases of each virus group using AlphaFold2-predicted structures. E- β strand (blue), L- loop (green), H- α helix (red). NCLDVs representative- Vaccinia virus A32 (yellow), membrane-containing dsDNA viruses’ representative- PRD1 P9 (pink), ssDNA filamentous phages representative- M13 gp1 (orange) (c) secondary structure elements and domain organization of representative viral ATPases. WA- Walker A motif, WB- Walker B motif, R- arginine finger, Q- sensor residue (red), TM- transmembrane domain (brown), CCM- coiled-coil motif (pink). Blue- β strands, yellow- α helices. Conserved ATPase core is highlighted in green. Scale bar: 10 aa. Accession no- Vaccinia virus: YP_233037.1, Organic Lake phycodnavirus 1: ADX05856.1, African swine fever virus: YP_009702812.1, M13 phage- NP_510893.1, PRD1 phage- AAX45927.1, FtsK_CΔγ_ PDB ID- 2IUT.

Bacterial FtsK is a dsDNA translocase involved in chromosome segregation during cell division. It comprises of three distinct domains- an N-terminal transmembrane domain responsible for localization and recruitment of accessory proteins, a C-terminal motor domain forming a RecA-like ATP hydrolysis/nucleotide-binding fold and an intervening linker domain rich in proline and glutamine residues [15, 16]. The viral putative genome packaging ATPases of filamentous phages (*Inoviridae* family), membrane containing dsDNA phages, and NCLDVs including poxviruses show the characteristic conservation of Walker A and Walker B motifs, arginine finger and sensor motif, specific to the Ftsk/HerA superfamily [2]. These proteins also exhibit strong structural conservation, despite their highly divergent protein sequences (Fig 1A and 1B). Computationally predicted secondary and tertiary structure analyses demonstrate that the viral proteins are much shorter and largely correspond only to the motor domain of their bacterial homolog i.e., FtsK_CΔγ_, with few variations like the presence of coiled-coil motif towards the C-terminus of poxviral ATPase or in the region between Walker A and Walker B motifs of *Asfarviridae* and *Phycodnaviridae*, and distinct transmembrane, cytoplasmic, and extracellular domains in filamentous phages (Fig 1C). They invariably form a RecA-like conserved β-sheet core with alternating α-helices, similar to bacterial FtsK (Fig A in S1 File). Thus, the virus-encoded putative genome packaging ATPases could potentially represent the minimal conserved ATPase/translocase domain of the FtsK/HerA superfamily.

### Thioredoxin tag promotes solubility and stability of the A32 protein

**Fig 2:**
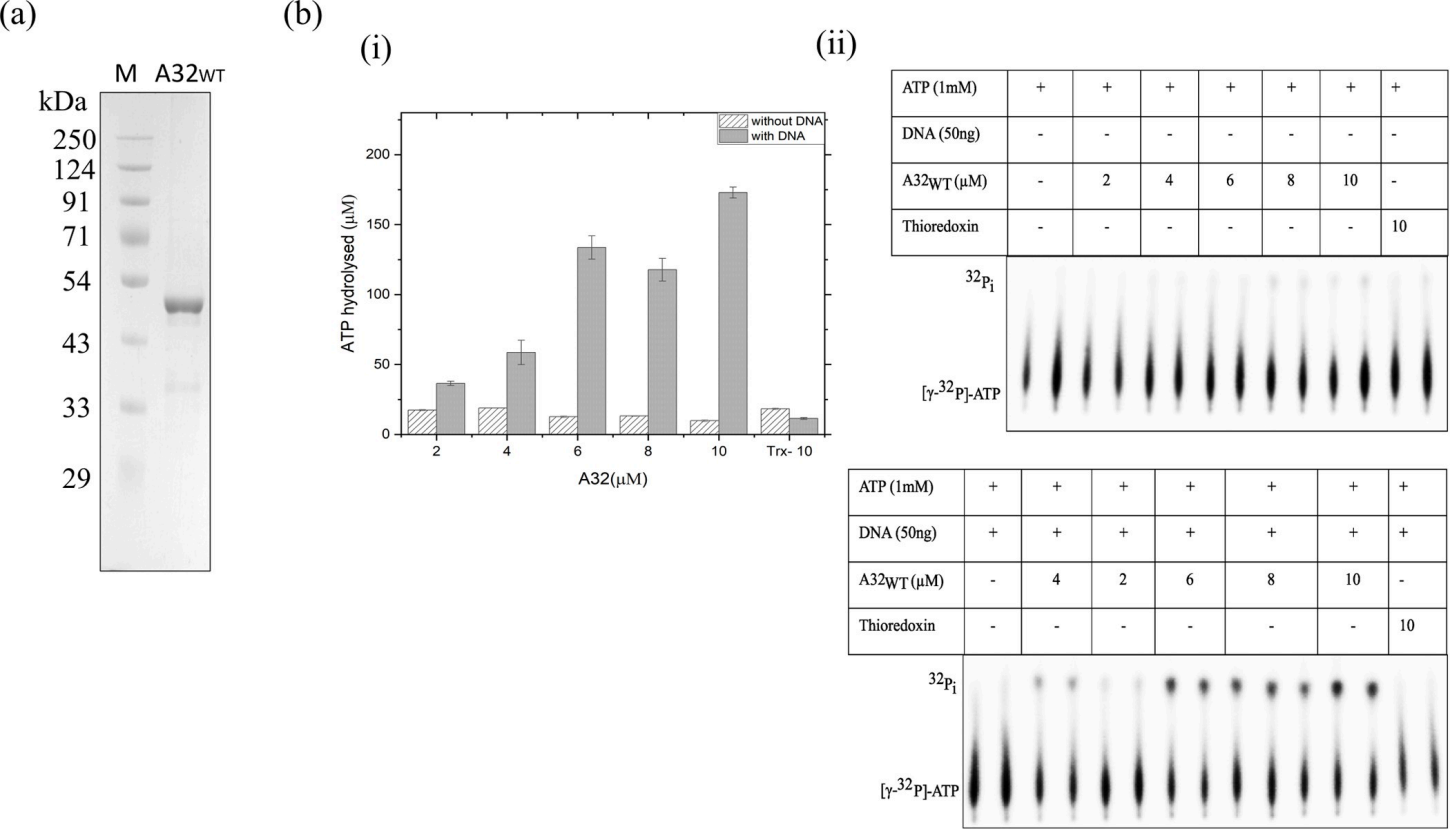
Purification and ATPase activity of A32_WT_. (a) Purified A32_WT_ protein. M-marker, A32_WT_- Purified and concentrated A32 protein after Heparin and Ni^2+^-NTA affinity chromatography (b) (i) bar graph and (ii) autoradiograph showing ATPase activity in the presence/absence of DNA as a function of A32_WT_ concentration. Trx-10 denotes 10 μM thioredoxin control. Values represent mean of duplicates with standard deviation, normalized with no protein control.

Several strategies were tested to obtain the purified A32 protein (summarized in Table 1). A32 protein expressed in *Escherichia coli* by the wildtype gene was highly insoluble and eluted along with many impurities, whereas the protein from its codon-optimized sequence showed negligible expression. Since A32 consists of 8 cysteine residues which might be involved in disulfide bond formation, its expression was also attempted in the *E*. *coli* periplasm, but to no success. Its expression in *E*. *coli* with an N-terminal GST-fusion resulted in possibly incorrect folding and tag auto-cleavage. Interestingly, A32 also did not express in the baculovirus-mediated insect cell expression system (Figs B-D in S1 File). A32 protein with an N-terminal fusion with thioredoxin tag and a C-terminal hexahistidine tag (A32_WT_), when expressed in *E*. *coli*, could be obtained in the soluble fraction and was successfully purified by Ni^2+^-NTA and heparin affinity chromatographies, albeit with few impurities (concentration of the purified protein was ~1 mg/ml). Furthermore, the ATPase activity of A32_WT_ protein increased by up to 17-fold in the presence of DNA at the highest protein concentration (Fig 2).

**Table 1:** Summary of various strategies tested to obtain purified A32 protein of vaccinia virus.

Feature	Vector	Observation/result
C-terminal 6x-Histidine tag, codon optimized for *E*. *coli* expression	pET28a	Low/no expression
C-terminal 8x-Histidine tag	pET41a	Low solubility, eluted with impurities
N-terminal Thioredoxin tag, C-terminal 6x-Histidine tag	pET32b	Low solubility, purified and used for ATPase activity analysis
Thioredoxin tag cleavage (enterokinase)	pET32b	Non-specific cleavage, protein precipitation
N-terminal Thioredoxin tag, C-terminal 6x-Histidine tag		
Thioredoxin tag cleavage (NT*-HRV3CP)	pET32b-HRV3C	Low solubility, truncation of protein
N-terminal Thioredoxin tag, C-terminal 6x-Histidine tag		
N-terminal GST tag	pGEX-6P-1	Low solubility, inefficient column binding, tag autocleavage
Periplasmic expression,	pET22b	No expression
N-terminal pelB sequence, C-terminal 6x-Histidine tag		
Baculovirus expression system	pFastBac1	No expression

We attempted to remove the thioredoxin tag by treating with enterokinase, but observed non-specific cleavage and protein precipitation at both 4° C and 20° C (Fig E in S1 File). When the enterokinase cleavage site (DDDDK) was replaced with the NT*-HRV3C cleavage site (LEVLFQGP) in the pET32b vector, the resultant protein (A32*_WT_) was highly insoluble. The concentrated, partially purified protein (~0.9 mg/ml) was treated with purified NT*-HRV3C protease at wt/wt ratios 1:1 and 1:2 A32*_WT_: NT*HRV3CP. Compared to no protease control, reduction in the concentration of A32*_WT_ was noted, whereas some protein remained uncut. Because of their similar sizes, NT*-HRV3C protease (35 kDa) and A32-6xHis (32 kDa) could not be distinguished on the SDS-PAGE gel. Western blot with anti-A32 immune sera showed a band around 16 kDa, indicating non-specific cleavage or truncation of the protein upon removal of the thioredoxin tag (Fig E in S1 File).

Thus, we demonstrate that pure and functional A32 protein could only be obtained when fused with the thioredoxin tag.

### A32 forms a conserved coiled-coil motif towards its C-terminus

**Fig 3:**
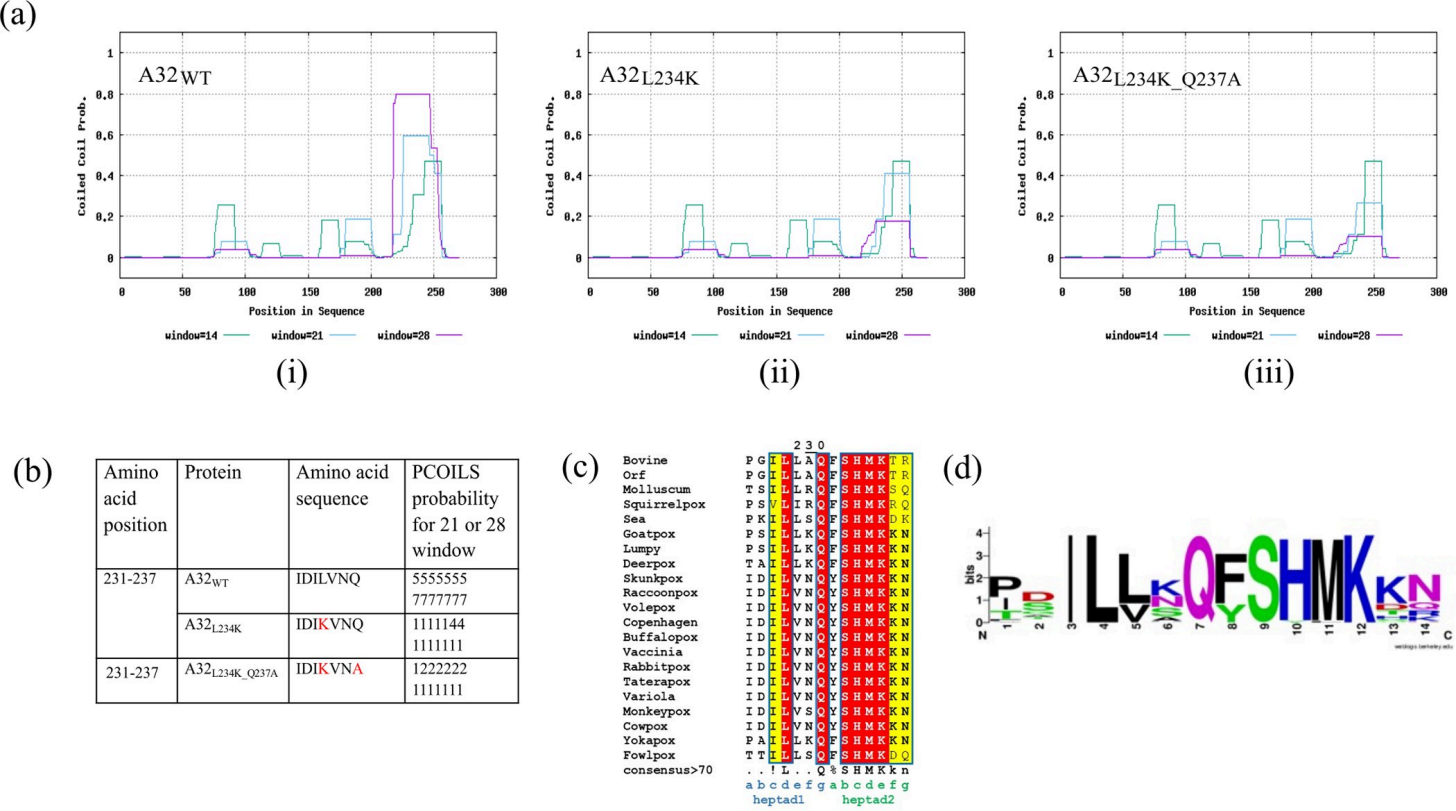
Assessment of coiled-coil motif in A32. (a) Probability plot for coiled-coil motif prediction by PCOILS in (i) A32_WT_ (ii) A32_L234K_ and (iii) A32_L234K_Q237A_. The default output of probabilities in the scanning windows of 14 (green), 21 (blue) and 28 (purple) aa residues are shown. (b) The predicted probability of heptad repeats between aa residue 231 to 237 for the scanning windows of 21 or 28. (c) Multiple sequence alignment of coiled-coil region in A32 homologs of *Chordopoxvirinae* subfamily using MUSCLE. Red- strictly conserved residues; yellow-conserved amino acids in majority of the sequences (d) Bit map image for the conservation of coiled-coil motif in *Chordopoxvirinae*.

A coiled-coil motif (CCM) was predicted in the C-terminal region of A32 between aa 231 to 250. With the hypothesis that substitution of conserved hydrophobic aa residue(s) with hydrophilic aa might destabilize the coiled-coil motif, A32 mutants- A32_L234K_ and A32_L234K_Q237A_ were designed. Both mutants show much reduced probability to form the CCM as predicted by the PCOILS server. The CCM motif selected for mutagenesis was also found to be strictly conserved in other poxvirus family members (Fig 3).

### Coiled-coil motif regulates the DNA-dependent ATPase activity of A32

**Fig 4:**
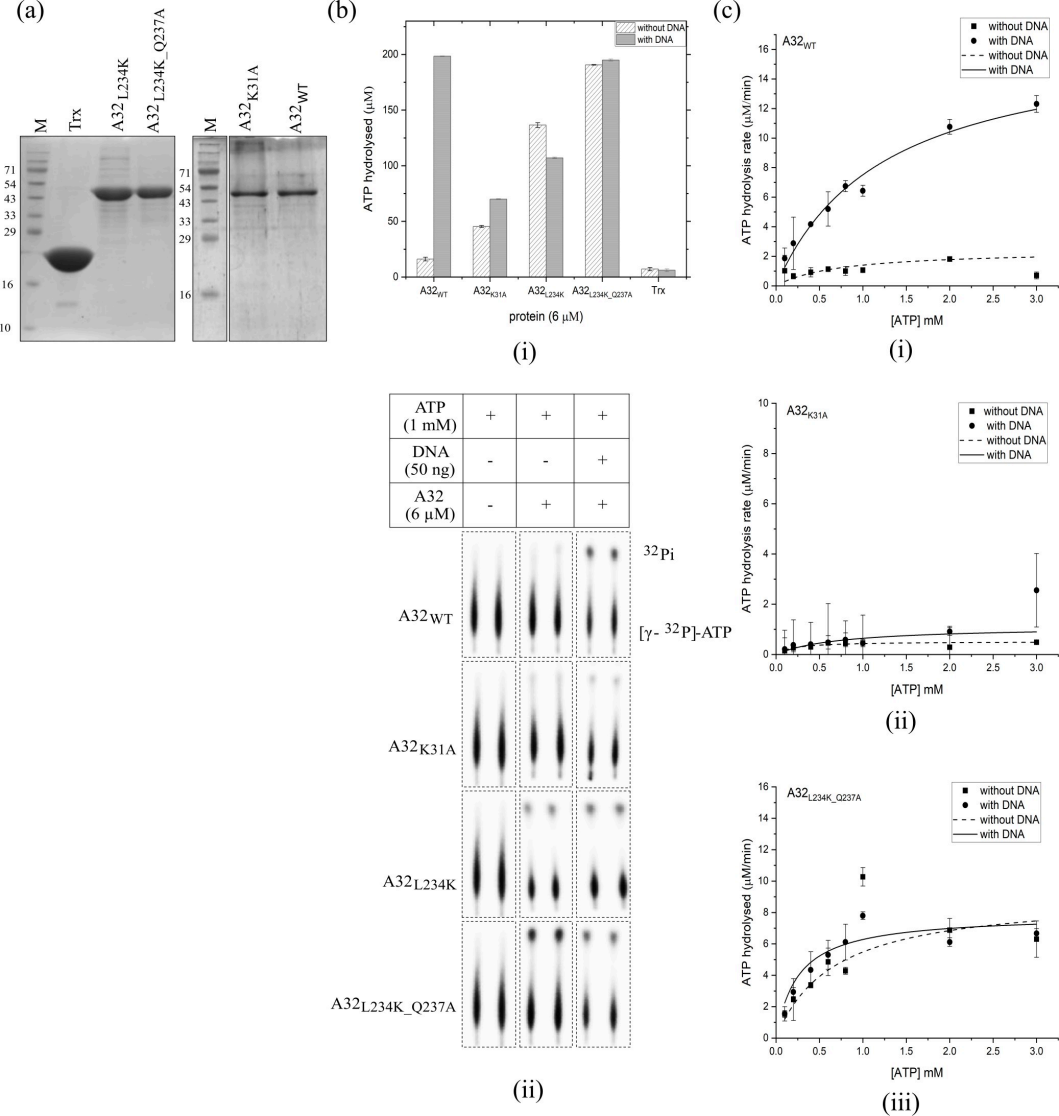
Regulation of ATPase activity by coiled-coil motif (a) Purified proteins. Trx- Thioredoxin, A32_WT_- A32 wild type, A32_K31A_- Walker A motif mutant, A32_L234K_- coiled-coil motif mutant 1, A32_L234K_Q237A_ - coiled-coil motif mutant 2, M- marker. (b) (i) bar graph and (ii) autoradiograph of comparative ATPase activities of wildtype A32_WT_ and its mutants. Trx denotes 10 μM thioredoxin control. Lanes from original image have been rearranged for representation. Values represent mean of duplicates with standard deviation, normalized with no protein control. (c) Steady- state kinetics analysis of (i) A32_WT_ (ii) A32_K31A_ and (iii) A32_L234K_Q237A_. Values represent mean of duplicates with standard deviation, normalized with no ATP control.

In addition to the CCM mutants A32_L234K_ and A32_L234K_Q237A_, a Walker A lysine mutant (A32_K31A_) that should abolish the ATPase activity [17, 18], was also generated. Similar to the wildtype A32_WT_, all three mutants comprise N-terminal thioredoxin tag and C-terminal hexahistidine tag. They were purified by Ni^2+^-NTA and heparin affinity chromatographies and concentrated to about 0.7–1 mg/ml. Purified wildtype and mutant proteins contained some impurities along with the protein of interest (Fig 4A).

A comparative analysis of the ATPase activities showed that unlike the wildtype A32_WT_, the Walker A mutant A32_K31A_ possesses negligible activity with or without DNA. This loss of activity confirms that the observed ATPase activity of A32_WT_ could indeed be attributed to the A32 protein and not the co-purified impurities. Interestingly, the CCM mutants A32_L234K_ and A32_L234K_Q237A_ showed enhanced basal ATPase activities. While the ATP binding and hydrolysis activities were retained in these mutants, the DNA dependence of their ATPase activity was abolished (Fig 4B). Thus, the disruption of the CCM of A32 protein resulted in the loss of DNA-dependent-regulation of ATPase activity observed in the wildtype protein. These observations were further confirmed by carrying out steady-state kinetics analysis with trace amounts (~ 50 nM) of radioactive [γ-^32^P]-ATP and varying concentrations of non-radioactive ATP (Fig 4C and Fig F in S1 File). The V_max_ and K_cat_ calculated for A32_L234K_Q237A_ were similar in the absence or presence of DNA, and approximately half the value compared to A32_WT_ in the presence of DNA (Table 2).

**Table 2:** Steady-state kinetic parameters obtained for ATPase activity of A32 and its mutants.

Kinetic Parameters	A32_WT_		A32_K31A_		A32L_L234K_Q237A_	
	-	+	-	+	-	+
	DNA	DNA	DNA	DNA	DNA	DNA
V_max_ (μM min^-1^)	2.43	16.96	0.52	1.12	9.12	7.87
K_cat_ (min^-1^)	0.41	2.83	0.09	0.19	1.52	1.31
K_m_ (μM)	0.74	1.26	0.20	0.74	0.66	0.25

**Fig 5:**
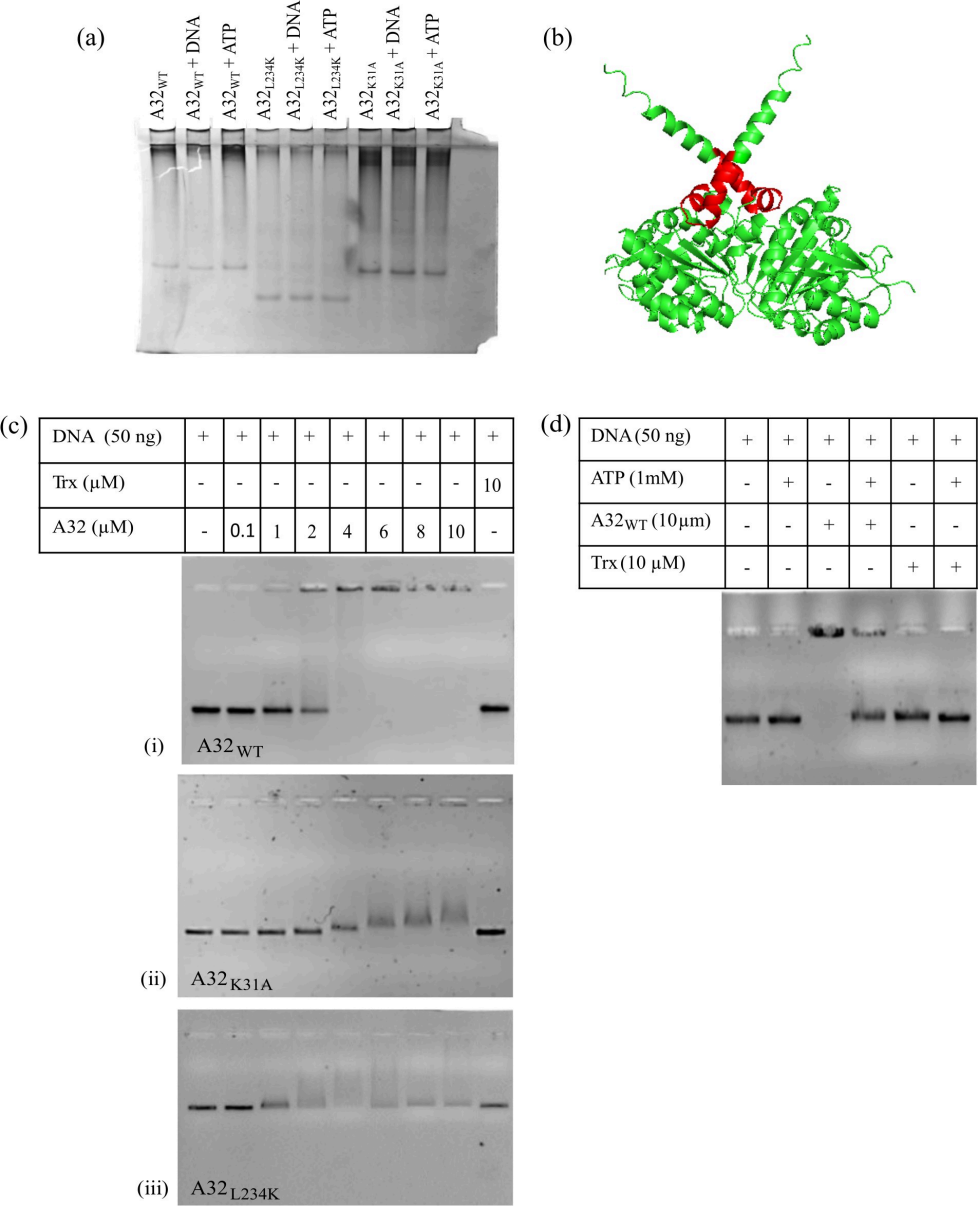
Higher order structure formation and DNA-binding by A32. (a) Native PAGE comparison of A32 or its mutants in the presence or absence of linear dsDNA or ATP. (b) AlphaFold2-predicted dimeric structure of A32, with the predicted coiled-coil motif highlighted in red. (c) EMSA with increasing concentration of (i) wildtype A32_WT_ (ii) Walker A motif mutant A32_K31A_ and (iii) CCM mutant A32_L234K_. (d) EMSA of 10 μM wildtype A32_WT_ in the presence or absence of 1 mM ATP. Trx denotes 10 μM purified thioredoxin control.

Coiled-coil motif has been implicated to play a role in protein oligomerization [19, 20]. Native PAGE analysis of purified proteins demonstrates the inability of only the CCM mutant, but not the Walker A mutant, to form higher order structure (Fig 5A). A32 dimer structure predicted using AlphaFold2 suggests possible interaction between the A32 subunits via the coiled-coil motif (Fig 5B). Further, electrophoretic mobility shift assays (EMSA) demonstrate that the Walker A motif mutant A32_K31A_ and CCM mutant A32_L234K_ do not bind to DNA as efficiently as the A32_WT_. Interestingly, upon ATP addition, the protein-bound DNA reduces, and free DNA is released (Fig 5C and 5D). The results together indicate that A32 oligomerization is not dependent on DNA or ATP binding, however, binding of A32 to DNA requires the protein to be in its oligomeric form with intact ATP-binding-Walker A motif. Since ATP hydrolysis is enhanced upon DNA binding, we believe that protein oligomerization, mediated by the coiled-coil motif, regulates the ATPase activity of A32. We speculate whether binding of DNA to the ATP-bound-A32 results in ATP hydrolysis, which in turn may lead to conformational changes in the oligomeric A32 and its reduced affinity towards the bound DNA. Thus, the coupling of ATPase regulation to DNA- binding and oligomerization of the A32 protein appears to have mechanistic relevance and calls for further investigations.

## Conclusion

Putative genome packaging ATPases of NCLDVs, ssDNA filamentous phages and membrane-containing dsDNA phages, despite their low sequence similarity, share a highly conserved structural fold characteristic of the FtsK/HerA superfamily [2] with few additional motifs in some viruses. The A32 structure was predicted with high confidence (average pLDDT score = 89.8) using AlphaFold2 except the C-terminal helix and the N-terminus. Our sequence and secondary structure analyses of the poxvirus A32 putative genome packaging ATPase showed the presence of a conserved coiled-coil motif (CCM) towards its C-terminal end. The CCM has also been reported in the hexameric bacterial FtsK protein [2]; however, its role in the protein’s activity has not been implicated. To discern if the CCM has any influence on the ATPase activity of putative genomic packaging ATPase, we purified and compared the activity of thioredoxin-tagged A32 wildtype protein with the thioredoxin-tagged CCM mutants. Our results show that the ATPase activity of A32_WT_ protein is stimulated in the presence of DNA. This finding is consistent with the outcomes reported for A32 homologs of Goatpox virus [11] and Orf virus [12]. This DNA-dependence of its activity might be regulated by the CCM responsible for protein oligomerization, as evidenced by the observations that mutations disrupting the CCM motif led to ATP hydrolysis both in the presence and absence of DNA, but no higher order A32 formation or DNA binding. Dimeric A32 structure, generated using AlphaFold2 indicates the involvement of CCM in A32 oligomerization. Further biochemical and structural studies are needed to understand the mechanistic basis of this regulation, explore the protein’s stoichiometry and how the ATP and DNA bind to A32 and impact its folding; thereby, resulting in the ATP hydrolysis and possibly DNA translocation. Such studies should preferably be conducted with the native protein, devoid of any large tags like the ~17 kDa thioredoxin tag fused at the N-terminus of A32_WT_. However, our attempts to obtain pure and untagged A32 by various approaches indicate that in the absence of the thioredoxin tag, the protein is highly insoluble or prone to precipitation and truncations upon tag cleavage. More robust techniques must be employed to produce untagged A32 protein suitable for in-depth characterization of its structure and function. Hence, our study also provides opportunities for researchers to explore novel methods for soluble expression of the genome packaging ATPases of NCLDVs.

## Materials and methods

### Sequence and structure analysis

Vaccinia virus putative genome packaging ATPase, *A32L* (Gene ID: 3707685) sequence was retrieved from NCBI. Multiple sequence alignment was performed using Clustal omega [21]. Three-dimensional structures were predicted with the help of ColabFold v1.5.2-patch modification of AlphaFold2, using 6 recycles, no template and MSA generated using both Uniref and environmental databases [22, 23]. Transmembrane domains were predicted using DeepTMHMM [24] and Phobius [25]. Coiled-coil motif was predicted using PCOILS [26], NPS [27], and paircoil [28] servers. Experimental structure of *Pseudomonas aeruginosa* FtsKCΔγ was obtained from the PDB database (accession ID: 2IUT) [15]. Predicted ATPase structures of vaccinia virus A32, PRD1 P9, and M13 gp1 were aligned using US-Align [29]. All protein structure images were generated in PyMOL.

### Recombinant plasmids construction

#### Modification of pET32b plasmid

pET32b vector (Merck) was modified such that the enterokinase cleavage site was replaced with the human rhinovirus 14 3C protease (NT*-HRV3C protease) cleavage site (5’-CTGGAAGTTCTGTTCCAGGGGCCC-3’ coding for the peptide LEVLFQGP [30] through the reverse primer. Recombinant plasmid was constructed by PCR amplification of pET32b vector template using the forward primer, 5’-CCCCTCTAGAAATAATTTTGTTTAACTTTAAGAAGGAGATATACATATGAGC-3’ and the reverse primer, 5’-CCATGGCGGGCCCCTGGAACAGAACTTCCAGGGTACCCAGATCT-3’. The amplified PCR product was inserted into pET32b vector by the standard restriction-ligation cloning procedure. Replacement of protease cleavage site in the modified plasmid pET32b-HRV3C was confirmed by sequencing (Eurofins Genomics India Pvt. Ltd.).

#### Construction of A32L expression plasmids

*A32L* gene was PCR-amplified from the genomic DNA of vaccinia virus Western Reserve strain (ATCC^®^ VR-1354TM) and cloned into the bacterial expression vectors (Merck) pET41a, pET22b, pGEX-6P-1, pET32b, pET32b-HRV3C, and the Bac-to-Bac baculovirus expression system vector pFastBac1 (Invitrogen). A synthetic construct of *A32L* codon optimized for *E*. *coli*, *A32L*_*CO*_ (Biomatik), was cloned into the pET28a vector. All recombinant plasmids were constructed by standard restriction-ligation cloning methods using primers as described in Table A in S1 File. Walker A motif mutant (*A32L*_*K31A*_) and coiled-coil motif mutants (*A32L*_*L234K*_, *A32L*_*L234K_Q237A*_) were constructed by overlap PCR-based site directed mutagenesis using primers as described in Table B in S1 File and cloned into pET32b vector. Sequences of all recombinant constructs were verified by sequencing (Eurofins Genomics India Pvt. Ltd.).

#### Expression and purification of A32 protein

All recombinant constructs were transformed into competent *E*. *coli* BL21-CodonPlus (DE3)-RIPL cells (Agilent Technologies). Overnight grown primary inoculum was added to LB medium at 1% final concentration, grown at 37° C till O.D._600_ was 0.5–0.6, and induced with 0.5 mM IPTG at 20° C for 16 h. Induced cells were harvested, and cell pellet (except pET22b-*A32L*) resuspended in lysis buffer containing 50 mM Tris-HCl pH7.5, 400 mM NaCl, 10% glycerol, 1% triton X-100, 10 mM imidazole, 1 mM Benzamidine hydrochloride, and 1 mM PMSF. The suspension was treated with 1 mg/ml lysozyme at room temperature (25° C) for 20 minutes and cells lysed by sonication. Cell-free extract was obtained by centrifugation at 13,000 g and processed as follows for obtaining the pure protein.

#### Purification of A32 expressed from recombinant pET41a or A32_CO_ from recombinant pET28a vectors, respectively

The cell-free extract was incubated with Ni^2+^-NTA agarose beads (Genetix, India) pre-equilibrated with binding buffer (50 mM Tris-Cl pH 7.5, 400 mM NaCl, 10% glycerol, 20 mM imidazole, and 5 mM MgCl_2_) for 3 h. After washing the beads twice with the binding buffer, the protein of interest was eluted by resuspending them sequentially with elution buffer containing 100, 300, and 600 mM imidazole.

#### Purification of periplasmic A32 expressed from recombinant pET22b vector

*E*. *coli* periplasm was extracted by the cold- shock method described previously [31]. Briefly, cells were washed twice with 50 mM Tris-Cl pH 7.5, and resuspended in extraction buffer (0.2 M MgCl_2_, 20 mM Tris-Cl pH 7.5). Cell suspension was incubated in 35° C stirring water bath for 10 minutes followed by incubation on ice for 15 minutes. This was repeated twice. Periplasm was obtained by centrifugation at 20,000 g for 20 minutes at 4° C. Cell pellet was resuspended in 50 mM Tris-Cl pH 7.5 and lysed by sonication. Soluble cell-free extract and insoluble cellular debris and proteins were separated by centrifugation at 13,000 g. Periplasmic, cytoplasmic, and insoluble fractions were extracted from cells (-/+IPTG) transformed with pET22b or recombinant pET22b-*A32L* plasmids and analysed for A32 expression.

#### Purification of GST-tagged A32 expressed from recombinant pGEX-6P-1 vector

The cell-free extract was incubated with glutathione sepharose 4B beads (Cytiva) pre-equilibrated with binding buffer (50 mM Tris-Cl pH 7.5, 400 mM NaCl, 10% glycerol, 1 mM DTT) for 3 h. Flowthrough was collected and the beads washed twice with binding buffer. GST-tagged proteins were eluted with elution buffers containing 10 mM and 20 mM reduced glutathione.

#### Purification of thioredoxin-tagged A32 and its mutants expressed from recombinant pET32b or pET32b-HRV3C vectors

After separation of the cell-free extract, its NaCl concentration was adjusted to 200 mM and loaded onto HiTrap Heparin HP column (Cytiva) pre-equilibrated with binding buffer (50 mM Tris pH 7.4, 10% glycerol, and 200 mM NaCl). The protein was eluted by gradient elution (200 mM– 1 M NaCl). Peak fractions were pooled and loaded onto the Histrap HP column (Cytiva) pre-equilibrated with binding buffer containing 20 mM imidazole. The protein was eluted by gradient elution (20–600 mM). The pooled protein was concentrated, purity checked, and stored at -80°C. Walker A motif mutant A32_K31A_ was purified same as wild type A32 (A32_WT_). The coiled-coil motif mutants A32_L234K_ and A32_L234K_Q237A_ had reduced affinity for heparin, therefore, the mutants were first passed through the Histrap HP column and the eluates were pooled and passed through the Heparin column.

#### Cleavage of thioredoxin tag using enterokinase or NT*-HRV3C protease

35 μg purified thioredoxin tagged A32_WT_ protein (expressed from pET32b vector) was treated with 1 unit of Enterokinase (Novagen, Merck). Protease activity was monitored at 14, 17, and 36 h at 4° C or 20° C.

Recombinant construct expressing the NT*- HRV3C protease was obtained from Addgene and the protease purified by Ni^2+^-NTA affinity chromatography and size exclusion chromatography as previously described [30]. Partially purified A32*_WT_ protein (expressed from pET32b-HRV3C vector) was incubated with NT*-HRV3C protease at 4° C. Protease activity was monitored at 0, 2 and 10 h with either no protease, or in the presence of protease at 1:1, and 1:2 wt/wt ratios (A32*_WT_: NT*- HRV3C).

#### Expression of A32 protein in Sf9 insect cells using the Bac-to-Bac system

*A32L* gene was amplified and cloned into pFastBac1 vector (Invitrogen) followed by transformation into DH10Bac *E*. *coli* cells (Invitrogen). Transformed cells were identified by blue-white screening. Bacmids were purified using PureLink™ HiPure Plasmid Midiprep Kit (Invitrogen) according to the manufacturer’s protocol. ~5×10^5^ Sf9 cells (kind gift from Dr. Virupakshi Soppina, IIT Gandhinagar) maintained in Sf-900™ II SFM serum free media (Gibco) at 27° C were transfected with 1 μg *A32L*-bacmid or empty-bacmid using 8 μl Cellfectin® II Reagent (Invitrogen) according to the manufacturer’s protocol. Cell size enlargement and lysis were seen 6 days post transfection. P_0_ stock of the obtained baculovirus was used to infect ~1–2 ×10^6^ sf9 cells in a T25 flask and the P_1_ viruses subsequently transferred to 3–4×10^6^ cells in a T75 flask to obtain P_2_ virus. 1ml P_2_ virus was used to infect a 30 ml suspension culture at 3×10^6^ cells/ml and incubated at 27° C with shaking at 90 rpm. Cells were harvested before lysis by centrifugation at 8000 g. Total protein was extracted by adding lysis buffer containing 20 mM HEPES pH 7.5, 200 mM NaCl, 5 mM MgCl_2_, 0.5% NP-40, 7% sucrose, 1x protease inhibitor cocktail (Merck). Cell lysate was analyzed in comparison to empty bacmid-infected sf9 cells for A32 expression.

#### ATPase assay

Purified A32_WT_ or its mutants (2–10 μM) were incubated at 37° C for 30 minutes in a reaction mixture containing 1 mM non-radioactive ATP and trace amounts (~ 50 nM) of radioactive [γ-^32^P]-ATP (obtained from BARC, Mumbai), 50 mM Tris–HCl, pH 7.5, 0.1 M NaCl and 5 mM MgCl_2_ in the presence or absence of 50 ng DNA. Reaction was stopped with 50 mM EDTA followed by thin layer chromatography (TLC) (PEI-cellulose matrix, Sigma-Aldrich) and autoradiography. Steady-state kinetics was performed by varying the concentration of non-radioactive ATP from 0.1 to 3 mM, while keeping the concentration of [γ-^32^P]-ATP at ~ 50 nM and protein concentration at 6 μM. All reactions were performed in duplicates and the data points represent mean with standard deviations. Reproducibility was confirmed by repeating the experiment at least once under identical conditions. Imaging and quantification of the data was done by phosphor imaging using Typhoon FLA 9500 (GE Healthcare Life Sciences/Cytiva). Using the ImageQuant^TM^ software, the spot intensities in TLC images corresponding to ^32^P_i_ and [γ-^32^P]-ATP were estimated. From the ^32^P_i_ value, the total [P_i_] produced was calculated. The K_m_ and V_max_ values were determined by data fitting into the Michaelis–Menten equation using OriginPro 2023b software (Origin labs).

#### Native PAGE

Native PAGE was performed to compare the migration of purified wildtype A32_WT_, CCM mutant A32_L234K_, and the Walker A motif mutant A32_K31A_. 50 ng of 50 bp linear dsDNA or 1 mM ATP were used for incubation with A32 or its mutants. Samples were run on 5% polyacrylamide gel without SDS in Tris glycine buffer.

#### DNA-binding assay

Binding of A32 or its mutants to DNA was tested using the Electrophoretic Mobility Shift Assay (EMSA). Varying concentrations of A32 or its mutants, ranging from 0.1 μM to 10 μM, were incubated with 50 ng of linear 815 bp DNA, and ATPase assay buffer (20 mM Tris, 100 mM NaCl, 5 mM MgCl_2_) in a 10 μl reaction mixture at 30°C for 15 minutes. For experiment that required ATP, 1 mM ATP was added. Samples were run on 0.8% agarose gel in Tris-Boric Acid-EDTA buffer with 0.5 μg/ml ethidium bromide (EtBr), and analysed.

## Supporting information

S1 File Supporting tables and figures. Comprises Table A. List of primers used for A32L-recombinant plasmid construction; Table B. List of primers used for overlap PCR for construction of A32L mutants; Fig A. AlphaFold2 predicted three-dimensional structures of viral FtsK-like ATPases; Fig B. Expression of untagged A32 protein in *E*. *coli;* Fig C. Expression of A32 in recombinant baculovirus-infected sf9 cells; Fig D. Expression of GST-tagged A32 in *E*. *coli* cytoplasm; Fig E. Removal of thioredoxin tag of A32; Fig F. Autoradiographs of steady-state kinetics in the absence or presence of DNA.
(PDF)

S1 Raw image Raw gel and blot images. (PDF)

S1 Dataset Values used to build graphs. (XLSX)
