# Supplementary material for: Sequence and biochemical analysis of vaccinia virus A32 protein: Implications for in vitro stability and coiled-coil motif mediated regulation of the DNA-dependent ATPase activity
Source: PLoS One. 2025 Jan 6;20(1):e0316818. doi: 10.1371/journal.pone.0316818 (PMC11703096; doi:10.1371/journal.pone.0316818)
Supplement: S1 File — Comprises Table A. List of primers used for A32L-recombinant plasmid construction; Table B. List of primers used for overlap PCR for construction of A32L mutants; Fig A. AlphaFold2 predicted three-dimensional structures of viral FtsK-like ATPases; Fig B. Expression of untagged A32 protein in E. coli; Fig C. Expression of A32 in recombinant baculovirus-infected sf9 cells; Fig D. Expression of GST-tagged A32 in E. coli cytoplasm; Fig E. Removal of thioredoxin tag of A32; Fig F. Autoradiographs of steady-state kinetics in the absence or presence of DNA. (PDF) [file pone.0316818.s001.pdf]

Supporting tables and figures

Sequence and biochemical analysis of vaccinia virus A32 protein: Implications for *in vitro* stability and coiled-coil motif mediated regulation of the DNA-dependent ATPase activity

Uma Ramakrishnan[1¶], Tanvi Aggarwal[1¶] and Kiran Kondabagil[1*]

[1] Department of Biosciences and Bioengineering, Indian Institute of Technology Bombay, Powai, Mumbai, India

[*] Corresponding author

E-mail: kirankondabagil@gmail.com, kirankondabagil@iitb.ac.in (KK)

[¶] These authors contributed equally to this work.

Gene	Vector	Forward primer 5'→3'	Reverse primer 5'→3'
A32L	pET41a	ATCTACATATGATGAATTGTTTCCAAGAAAAACAA (NdeI)	ACAATACTCGAGTGATGATACATTTTTTGACGAC (XhoI)
	pET22b	ATAACACCATGGGCATGAATTGTTTCCAAGAAAAACAA (NcoI)	ACAATACTCGAGTGATGATACATTTTTTGACGAC (XhoI)
	pGEX-6P-1	ATCTGGGATCCATGAATTGTTTCCAAGAAAAACAA (BamHI)	ACTTAGTCGACTTATGATGATACATTTTTTGACGA (SalI)
	pET32b and pET32b-HRV3C	ATCCGCCATGGGCATGAATTGTTTCCAAGAAAAACAATTT (NcoI)	ACAATACTCGAGTGATGATACATTTTTTGACGAC (XhoI)
	pFastBac1	ATCTGGGATCCATGAATTGTTTCCAAGAAAAACAA (BamHI)	ACAATACTCGAGTTATGATGATACATTTTTTGACGAC (XhoI)
$A32L_{CO}$	pET28a	ATCTGCCATGGGCAACTGTTTCCAGGAAAAACAG (NcoI)	ACAATAAAGCTTACTACTAACATTTTTGCTGCTGC (HindIII)

Table A. List of primers used for A32L-recombinant plasmid construction. Restriction enzyme sites are underlined

Mutant	Overlap forward primer 5'→3'	Overlap reverse primer 5'→3'
$A32L_{K31A}$	TCTGGATCTGGAGCAACTATCTATTTA	TAAATAGATAGTTGCTCCAGATCCAGA
$A32L_{L234K}$	AACATCGATATTAAAGTAAATCAATATTC	GAATATTGATTTACTTTAATATCGATGTT
$A32L_{L234K\_Q237A}$	ATCGATATTAAAGTAAATGCATATTCGCAC	GTGCGAATATGCATTTACTTTAATATCGAT

Table B. List of primers used for overlap PCR for construction of A32L mutants. Modified codons are underlined

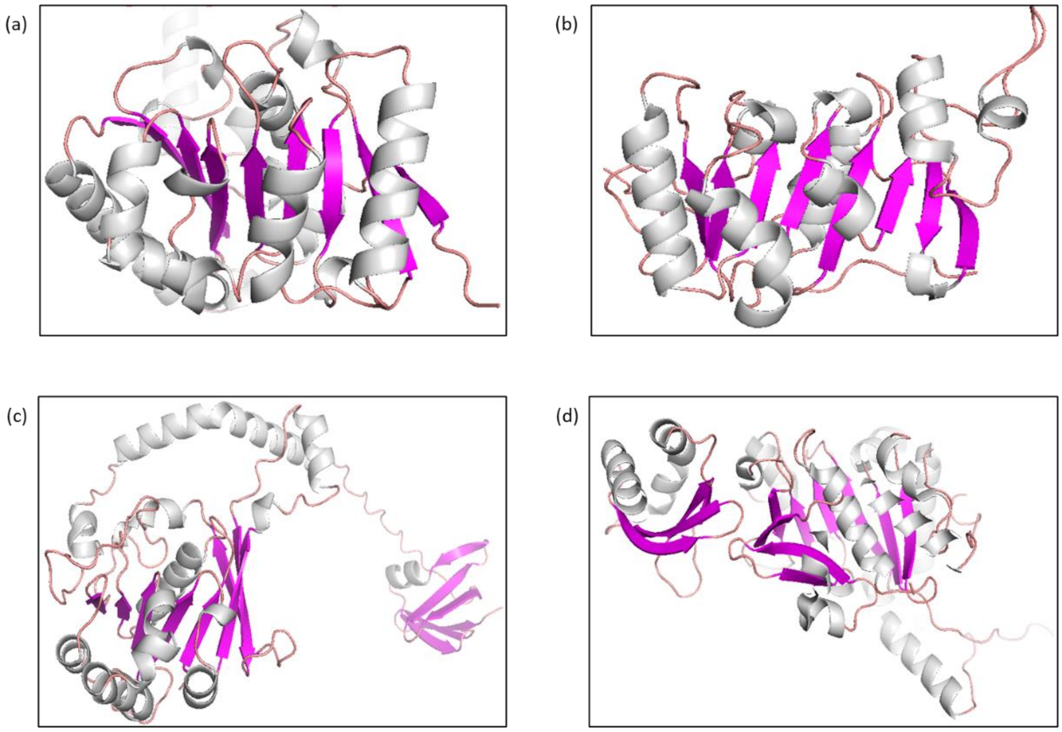

Fig A. AlphaFold2 predicted three-dimensional structures of viral FtsK-like ATPases. (a) Predicted structure of A32 protein of vaccinia virus (accession no. YP_233037.1) representative of NCLDVs (b) Predicted structure of P9 protein of PRD1 bacteriophage (accession no. AAX45927.1) representative of membrane-containing dsDNA bacteriophages (c) Predicted structure of gp1 protein of M13 bacteriophage (accession no. NP_510893.1) representative of ssDNA filamentous phages (d) Crystal structure of the motor domain of *P. aeruginosa* FtsK$_{C\Delta\gamma}$ (PDB ID- 2IUT). Conserved β-sheet core is highlighted in pink and α-helices are shown in grey.

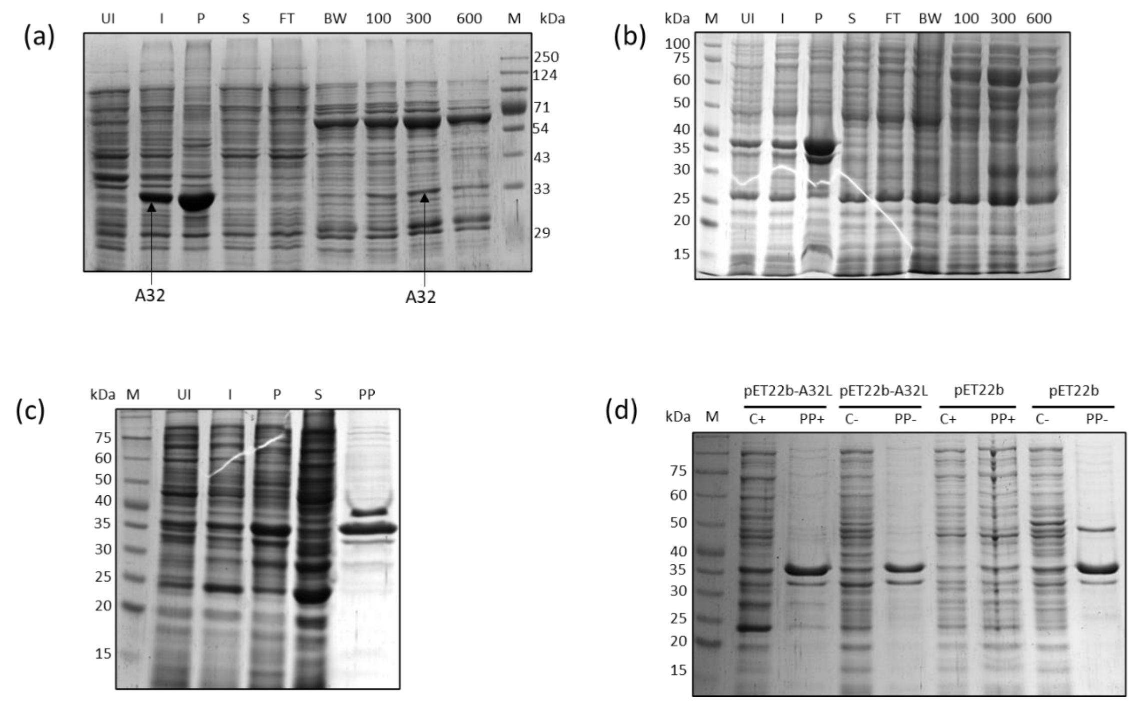

Fig B. Expression of untagged A32 protein in *E. coli* (a) cytoplasmic expression and Ni^{2+}-NTA resin binding of A32L cloned in pET41a vector (b) cytoplasmic expression and Ni^{2+}-NTA resin binding of codon-optimised $A32L_{CO}$ cloned in pET28a vector (c) Periplasmic expression of A32L cloned in pET22b vector (d) Periplasm extraction from *E. coli* cells. Cells were transformed with pET22b plasmid or recombinant pET22b-A32 plasmid and induced (+) or not induced (-) with IPTG. M-marker; UI-uninduced cell lysate; I-induced cell lysate; P-insoluble pellet; S-soluble cytoplasmic fraction; PP-periplasmic fraction; C- cell lysate; FT-Flow through; BW1-binding buffer wash 1; BW2-binding buffer wash 2; 100, 300, and 600-elution with 100, 300, and 600 mM imidazole, respectively

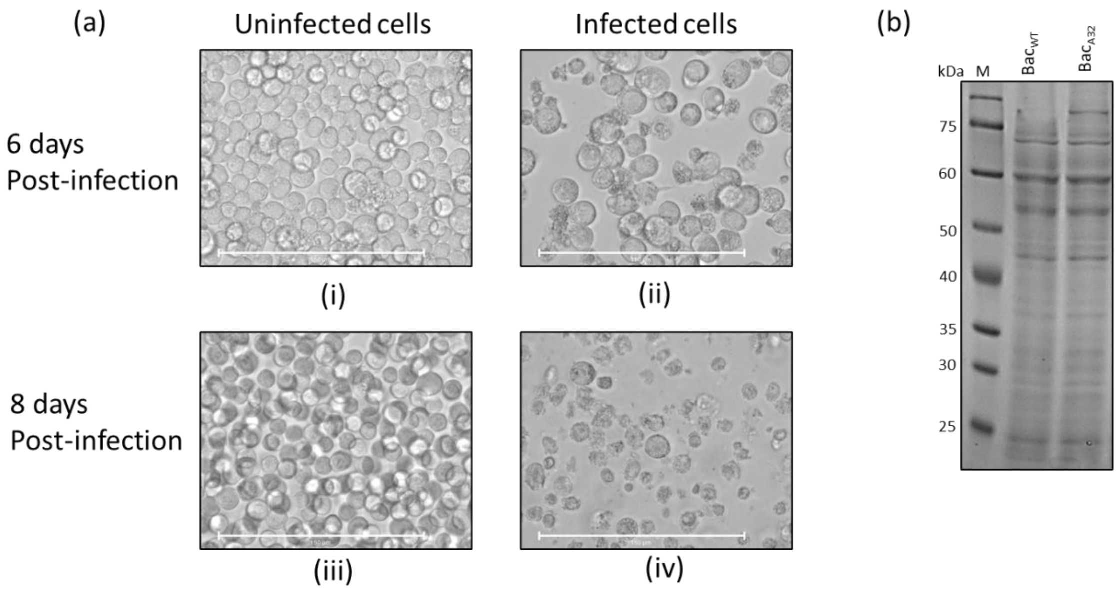

Fig C. Expression of A32 in recombinant baculovirus-infected sf9 cells. (a) Cytopathic effects of infecting sf9 cells with recombinant A32-baculovirus. (i) uninfected cells 6 dpi (ii) infected cells 6 dpi (iii) uninfected cells 8 dpi (iv) infected cells 8 dpi. Scale bar (white): 150 μM (b) SDS-PAGE analysis of sf9 cell lysate infected with baculoviruses. M-marker; Bac$_{WT}$-wildtype baculovirus; Bac$_{A32}$-recombinant baculovirus containing A32L gene

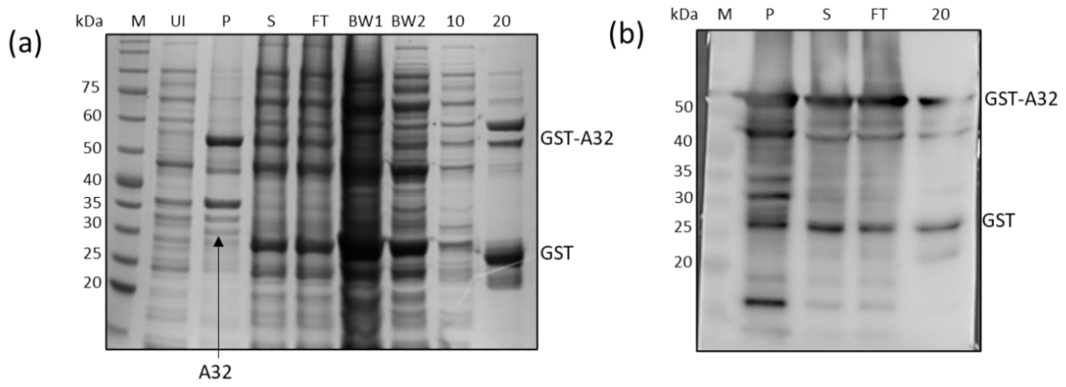

Fig D. Expression of GST-tagged A32 in *E. coli* cytoplasm (a) Expression and glutathione Sepharose bead binding of A32L cloned in pGEX-6P-1 vector (b) Western blot with anti-GST antibody. M-marker; UI- uninduced cell lysate; I-induced cell lysate; P-insoluble pellet; S-soluble protein supernatant; FT-unbound flow-through; BW1-binding buffer wash 1; BW2-binding buffer wash 2; 10 and 20-elution with 10 and 20 mM reduced glutathione, respectively

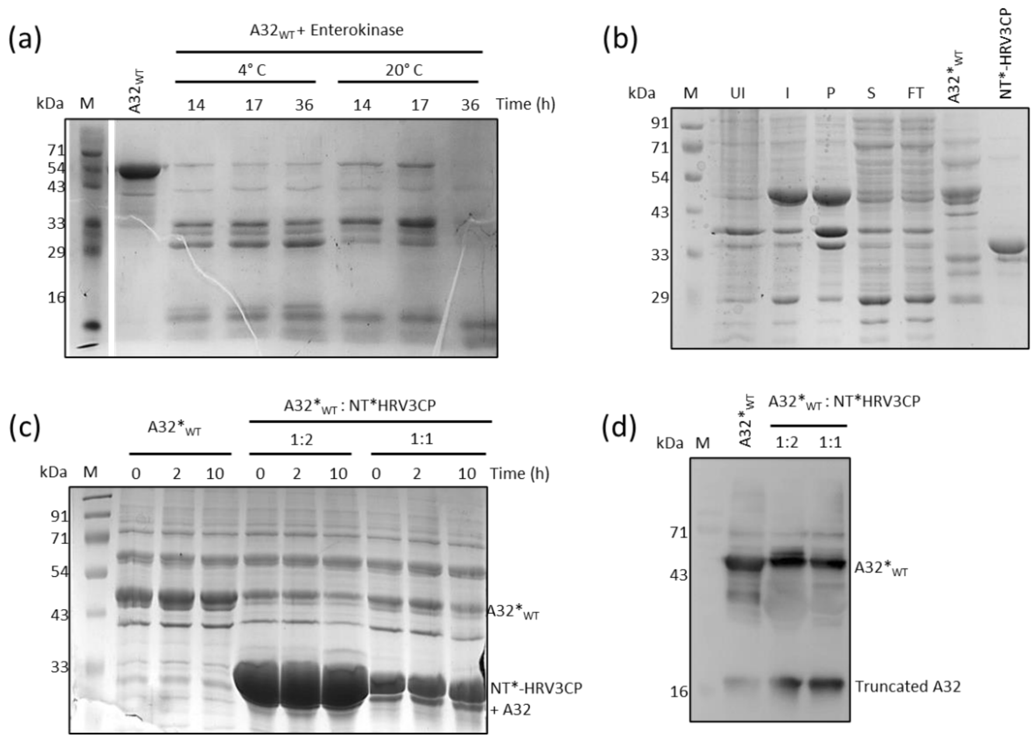

Fig E. Removal of thioredoxin tag of A32 (a) Cleavage of thioredoxin tag by enterokinase treatment. Thioredoxin tagged wild type A32 ($A32_{WT}$) was incubated with enterokinase at 4°C and 20°C for 14, 17, and 36 h (b) Expression and purification of $A32*_{WT}$ cloned in pET32b-HRV3C plasmid. M-marker; UI- uninduced cell lysate; I-induced cell lysate; P-insoluble pellet; S-soluble protein supernatant; FT-unbound flow-through; $A32*_{WT}$-partially purified by Ni^{2+} NTA chromatography; NT*-HRV3CP-purified protease (c) Cleavage of thioredoxin tag by NT*-HRV3CP treatment. $A32*_{WT}$ was incubated with NT*-HRV3CP protease at 1:2 and 1:1 wt/wt ratio for 0, 2 and 10 h at 4°C. (d) Western blot of $A32*_{WT}$ treated with protease for 10 h using anti-A32 immune sera

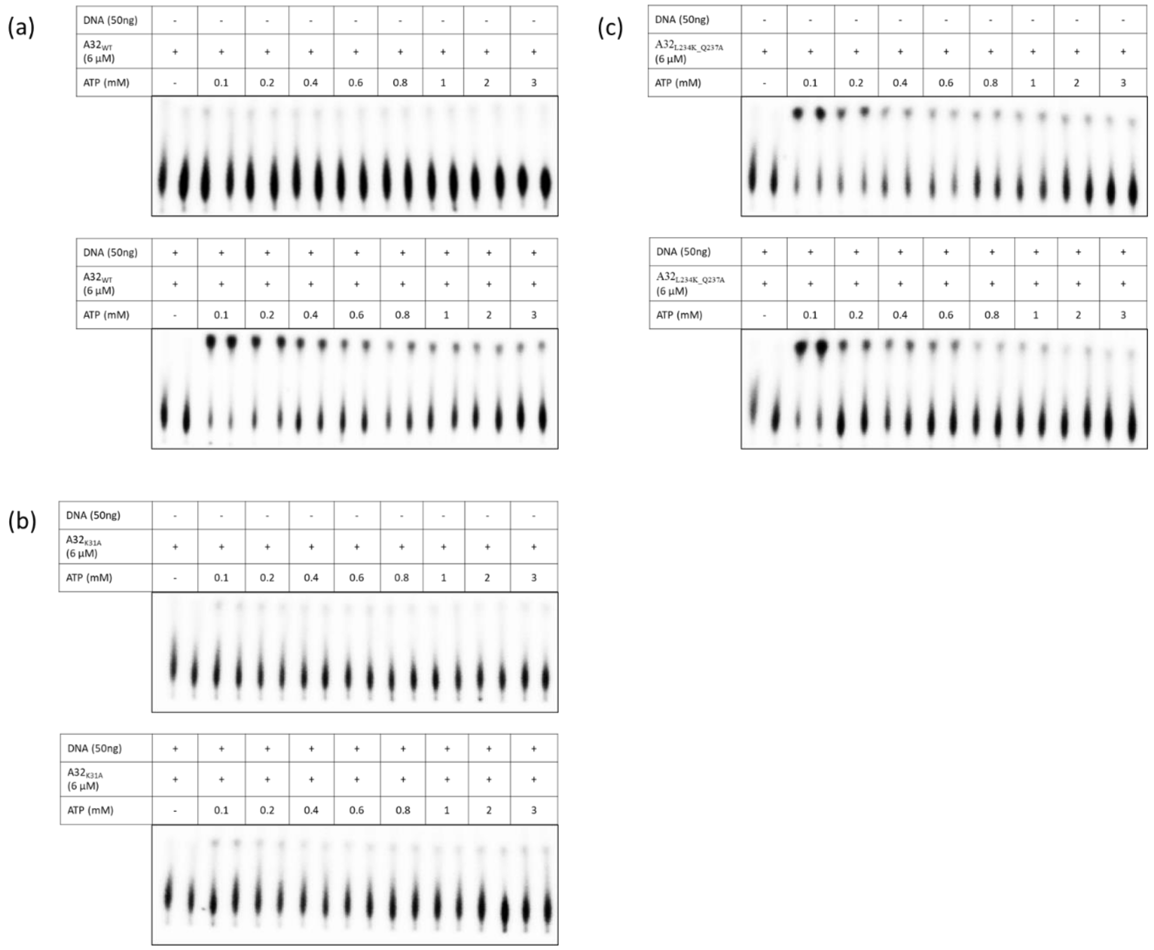

Fig F. Autoradiographs of steady-state kinetics in the absence or presence of DNA with (a) A32$_{WT}$, (b) Walker A motif mutant A32$_{K31A}$ and (c) coiled coil motif mutant A32$_{L234K_Q237A}$
