# Supplementary material for: Sequence and biochemical analysis of vaccinia virus A32 protein: Implications for in vitro stability and coiled-coil motif mediated regulation of the DNA-dependent ATPase activity
Source: PLoS One. 2025 Jan 6;20(1):e0316818. doi: 10.1371/journal.pone.0316818 (PMC11703096; doi:10.1371/journal.pone.0316818)
Supplement: S1 Raw image — (PDF) [file pone.0316818.s002.pdf]

Fig 2a- Expression and purification of $A32_{WT}$

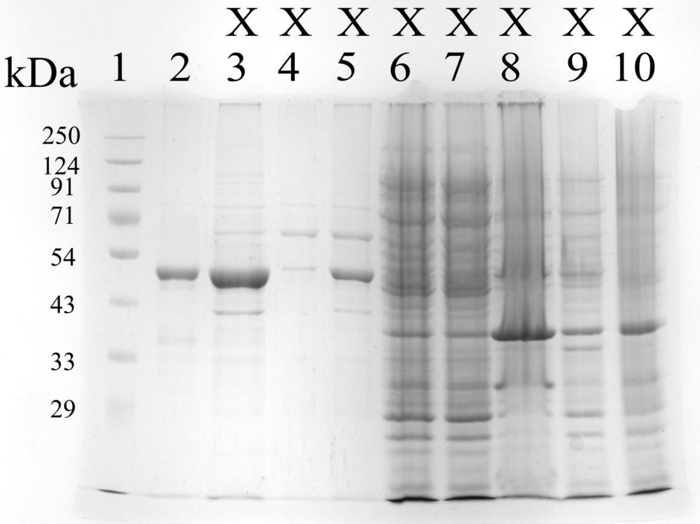

Loading order

1. Marker
2. Purified and concentrated Thioredoxin tagged A32 after Heparin and Histrap purification
3. Purified and pooled A32 after heparin column purification (sample for HisTrap purification)
4. Binding buffer wash- histrap purification
5. Flowthrough Histrap purification
6. Flowthrough Heparin column purification
7. Cell-free extract (soluble supernatant fraction)
8. Insoluble pellet (sample not loaded properly)
9. Induced cell lysate
10. Uninduced cell lysate

X- not included in the final image

Fig 2b.ii- Autoradiograph showing ATPase activity in the presence of DNA as a function of $A32_{WT}$ concentration

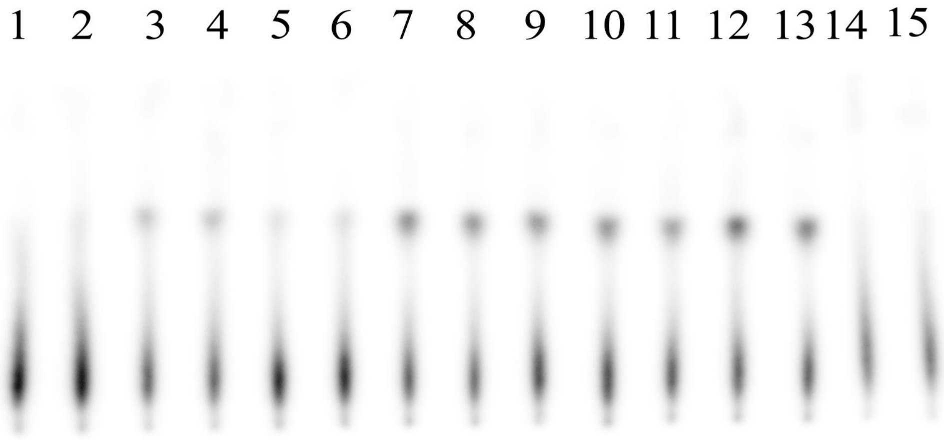

Loading order

1 and 2- no $A32_{WT}$
3 and 4- 4μM $A32_{WT}$
5 and 6- 2μM $A32_{WT}$
7 and 8- 6μM $A32_{WT}$
9,10 and 11- 8μM $A32_{WT}$
12 and 13- 10μM $A32_{WT}$
14 and 15- 10μM thioredoxin

Fig 2b.ii- Autoradiograph showing ATPase activity in the absence of DNA as a function of $A32_{WT}$ concentration

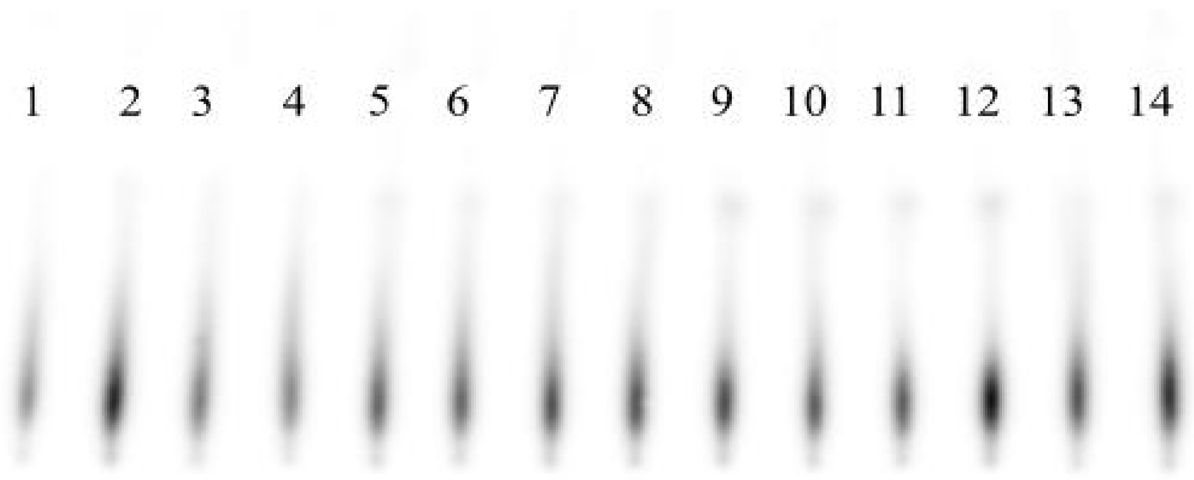

Loading order

1 and 2- no $A32_{WT}$
3 and 4- 2µM $A32_{WT}$
5 and 6- 4µM $A32_{WT}$
7 and 8- 6µM $A32_{WT}$
9 and 10- 8µM $A32_{WT}$
11 and 12- 10µM $A32_{WT}$
13 and 14- 10µM thioredoxin

Fig 4a. Purified proteins

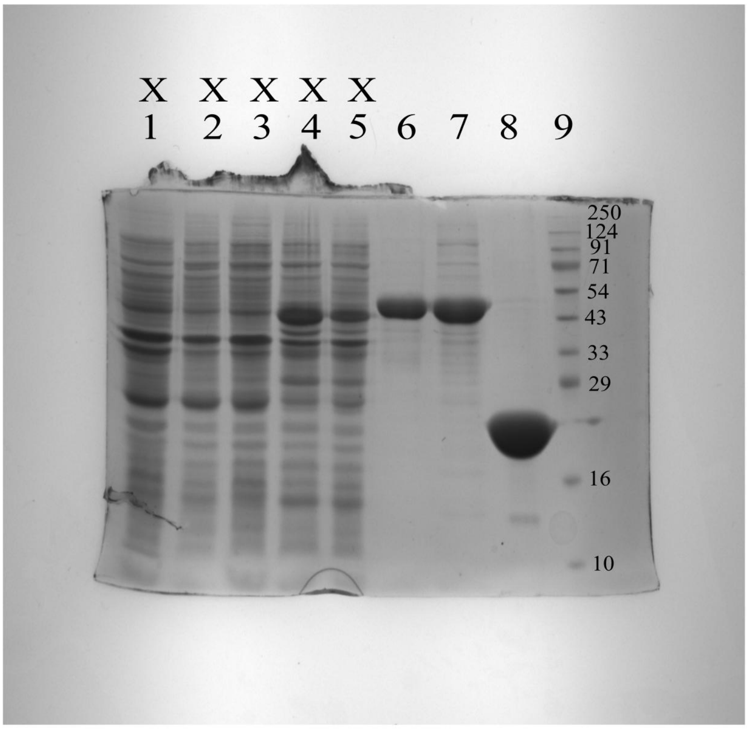

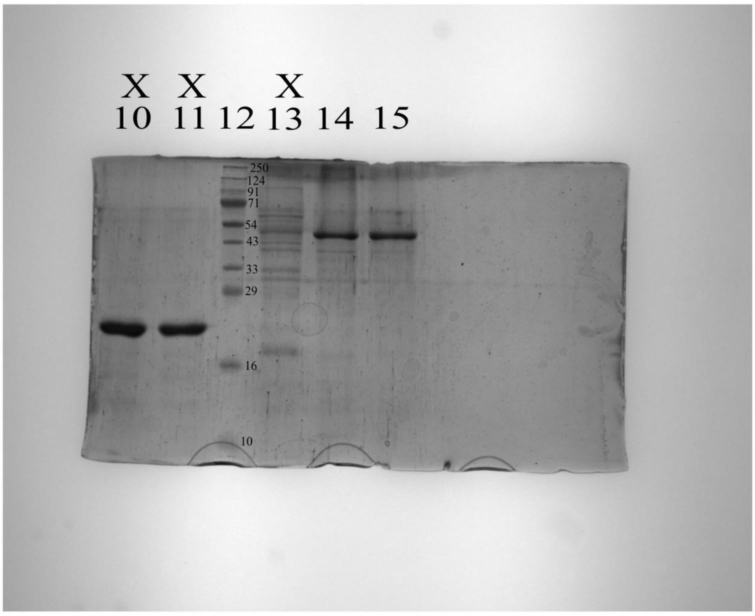

Loading order

1. Uninduced cell lysate- A32$_{L234K_Q237A}$
2. Uninduced cell lysate- A32$_{L234K}$
3. Uninduced cell lysate-thioredoxin
4. Induced cell lysate- A32$_{L234K_Q237A}$
5. Induced cell lysate- A32$_{L234K}$
6. Purified A32$_{L234K_Q237A}$
7. Purified A32$_{L234K}$
8. Purified Thioredoxin
9. Marker
10. Unrelated protein sample
11. Unrelated protein sample
12. Marker
13. Unrelated protein sample
14. A32$_{K31A}$
15. A32$_{WT}$

X- not included in final figure

Fig 4b.ii. Autoradiograph of comparative ATPase activities of wildtype A32$_{WT}$ and its mutants

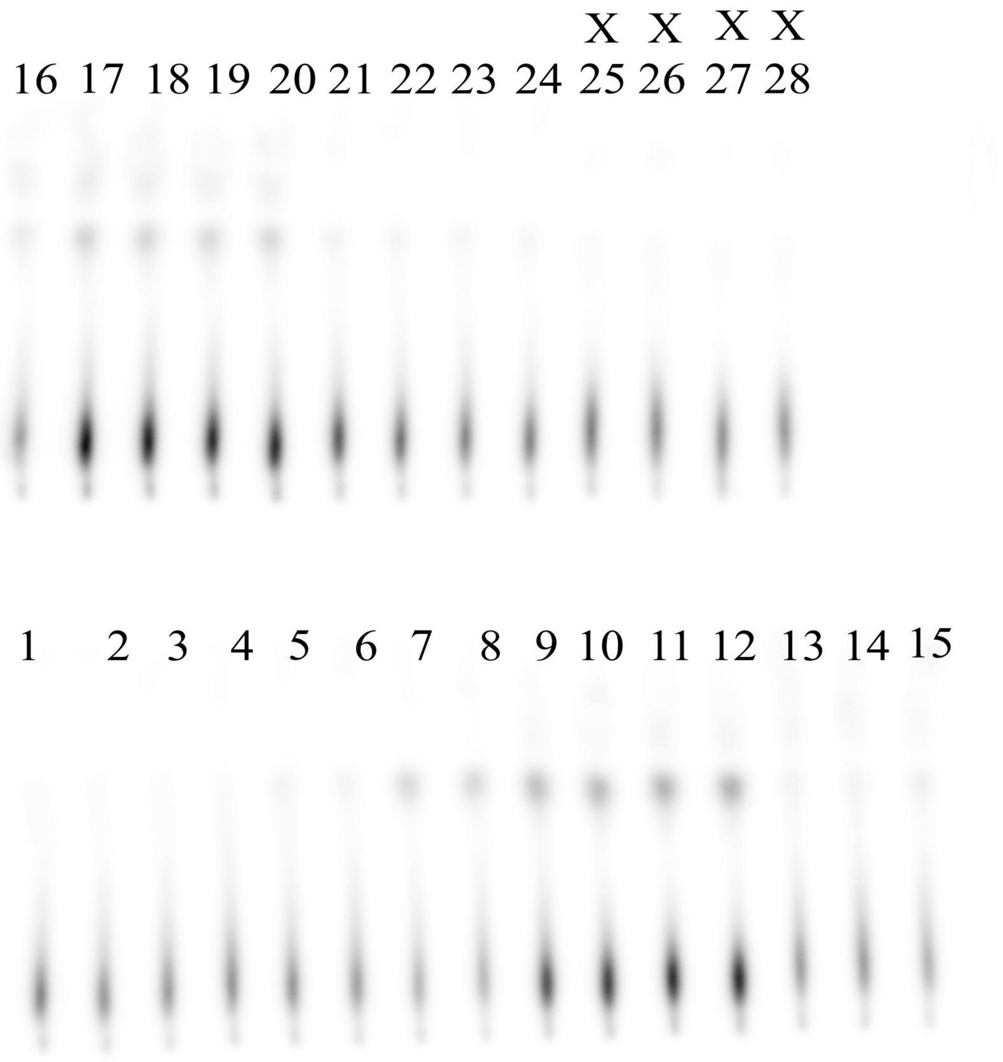

Loading order

1 and 2- ATP
3 and 4- ATP+ A32$_{WT}$
5 and 6- ATP+ DNA
7 and 8- ATP+ A32$_{WT}$ + DNA
9 and 10- ATP+ A32$_{L234K}$
11 and 12- ATP+ A32$_{L234K}$ +DNA
13 and 14- ATP+ A32$_{K31A}$
15 and 16- ATP+ A32$_{K31A}$ +DNA
17 and 18- ATP+ A32$_{L234K_Q237A}$
19 and 20- ATP+ A32$_{L234K_Q237A}$ +DNA
21 and 22- ATP+ A32$_{WT}$
23 and 24- ATP+ A32$_{WT}$ + DNA
25 and 26- ATP+ thioredoxin
27 and 28- ATP+ thioredoxin + DNA

X- not included in final figure

Fig 5a. Native PAGE of A32 and its mutants

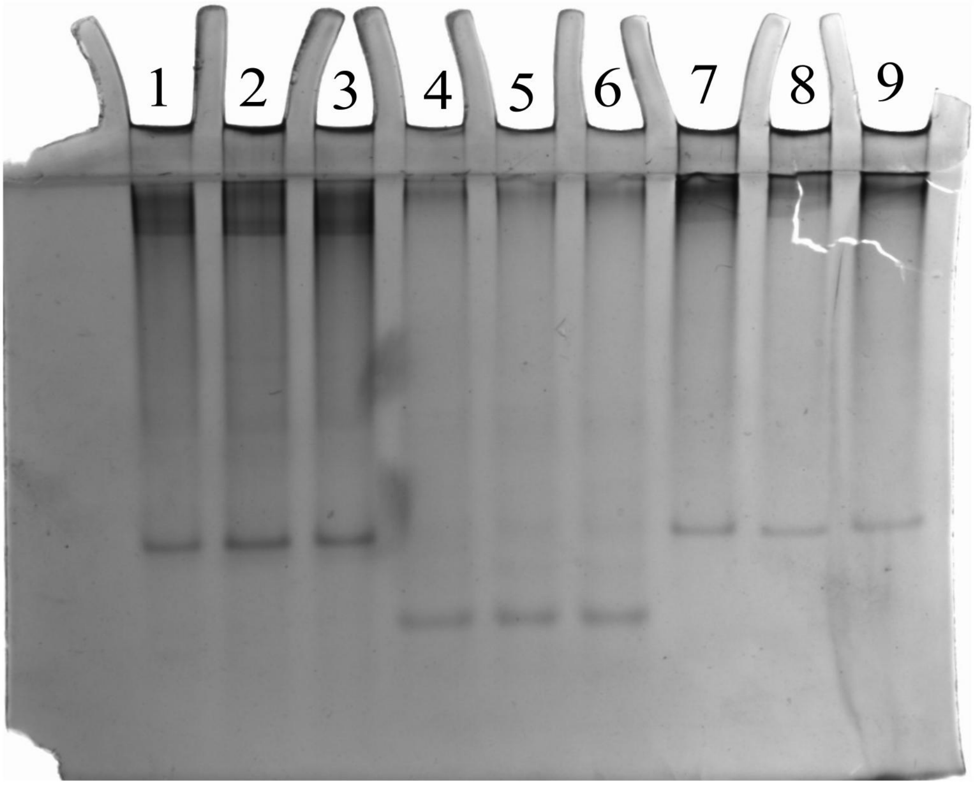

Loading order

1. $A32_{K31A}$ +ATP
2. $A32_{K31A}$ +DNA
3. $A32_{K31A}$
4. $A32_{L234K}$ +ATP
5. $A32_{L234K}$ +DNA
6. $A32_{L234K}$
7. $A32_{WT}$ +ATP
8. $A32_{WT}$ +DNA
9. $A32_{WT}$

Fig 5c. Electrophoretic mobility shift assays of A32 and its mutants

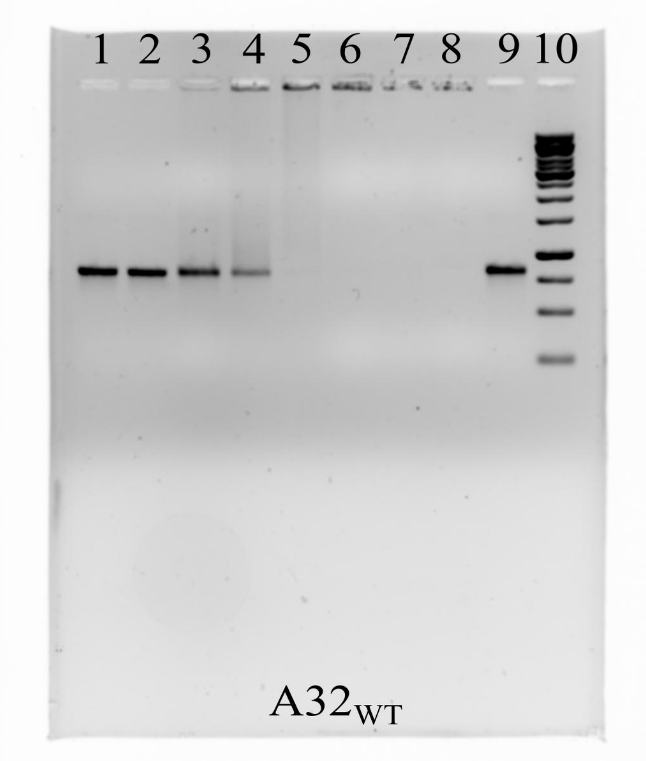

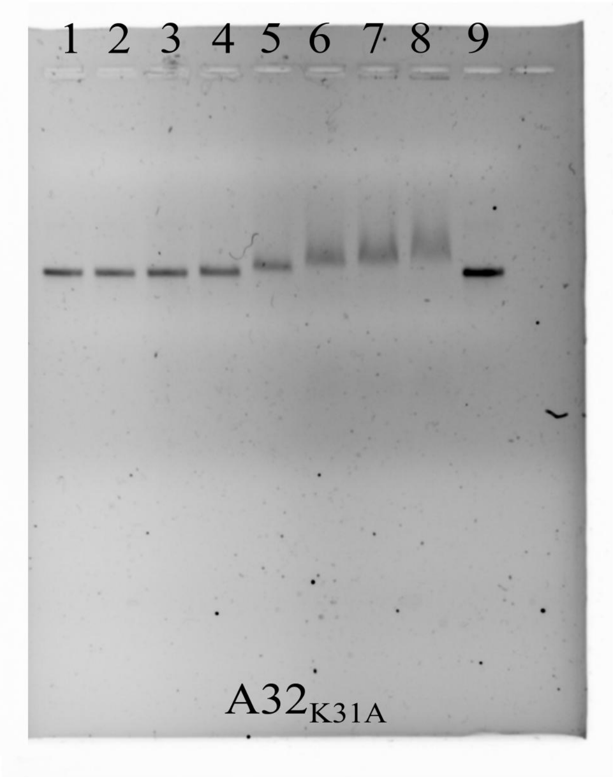

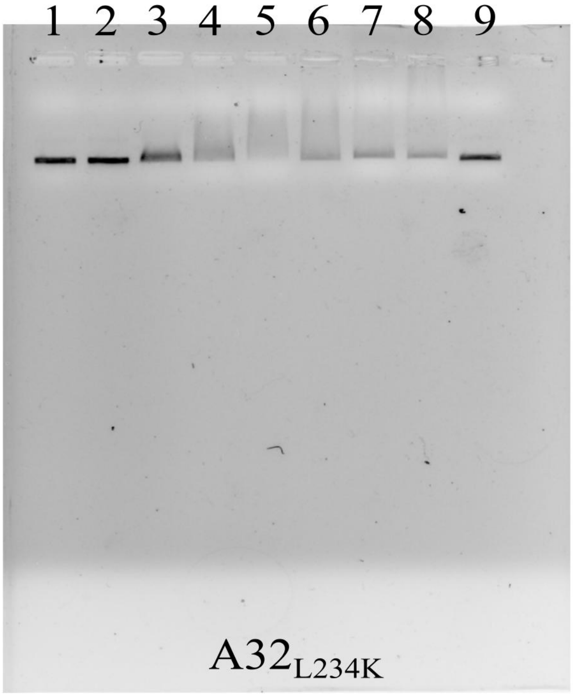

LANE	1	2	3	4	5	6	7	8	9	10
50 ng 815 bp Linear DNA	+	+	+	+	+	+	+	+	+	DNA ladder (1st gel)
A32 (μM)	-	0.1	1	2	4	6	8	10	-	-
Thioredoxin (μM)	-	-	-	-	-	-	-	-	10	-

Fig 5d. Electrophoretic mobility shift assay of $A32_{WT}$ in the presence of ATP

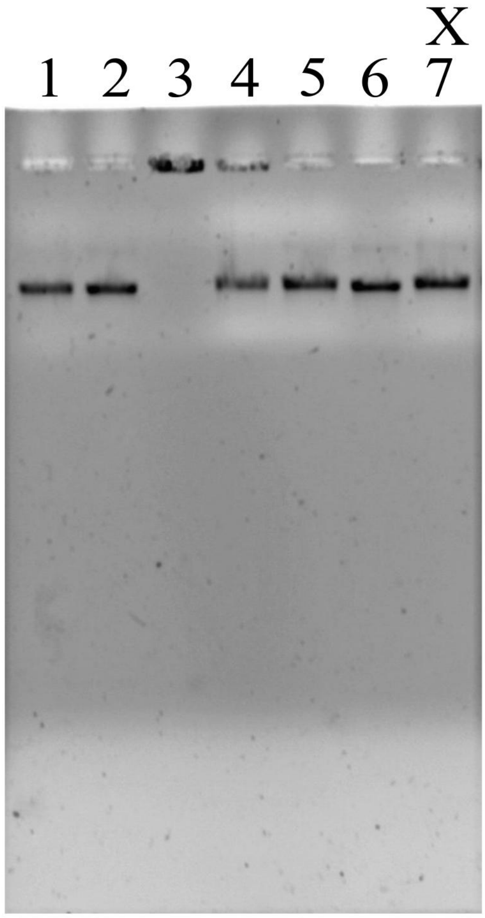

Loading order

1. 50 ng 815 bp linear dsDNA + assay buffer
2. Buffer + DNA + ATP
3. Buffer + DNA + $A32_{WT}$
4. Buffer + DNA + ATP + $A32_{WT}$
5. Buffer + DNA + thioredoxin
6. Buffer + DNA + thioredoxin + ATP
7. Buffer + DNA + ATP + $A32_{WT}$ (duplicate)

X- not included in final figure

S2a Fig. cytoplasmic expression and Ni^{2+}-NTA resin binding of A32L cloned in pET41a vector

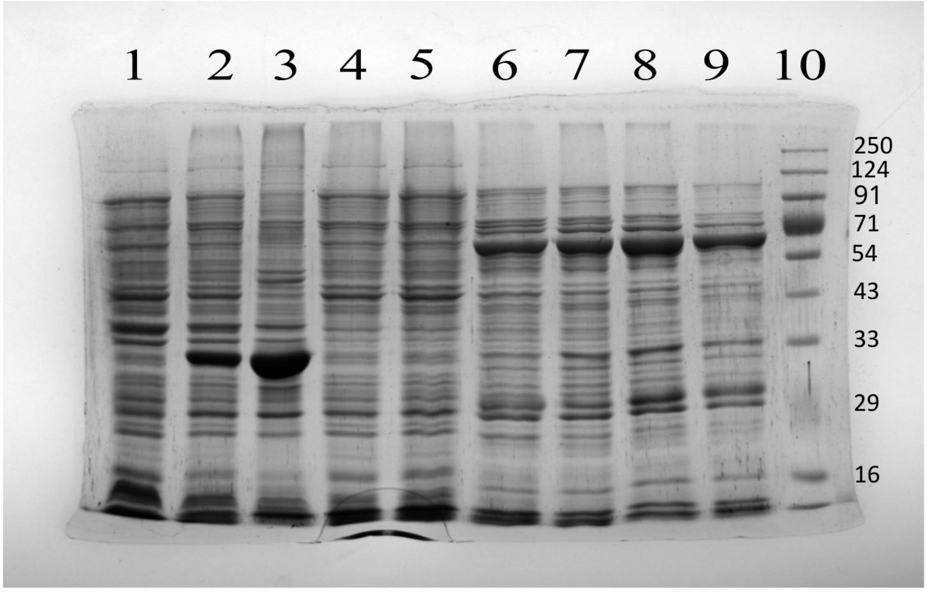

Loading order

1. Uninduced cell lysate
2. Induced cell lysate
3. Pellet
4. Supernatant
5. Flow through
6. 20mM imidazole wash
7. 100mM imidazole elution
8. 300mM imidazole elution
9. 600mM imidazole elution
10. Marker

S2b Fig. cytoplasmic expression and Ni^{2+}-NTA resin binding of codon-optimised $A32L_{CO}$ cloned in pET28a vector

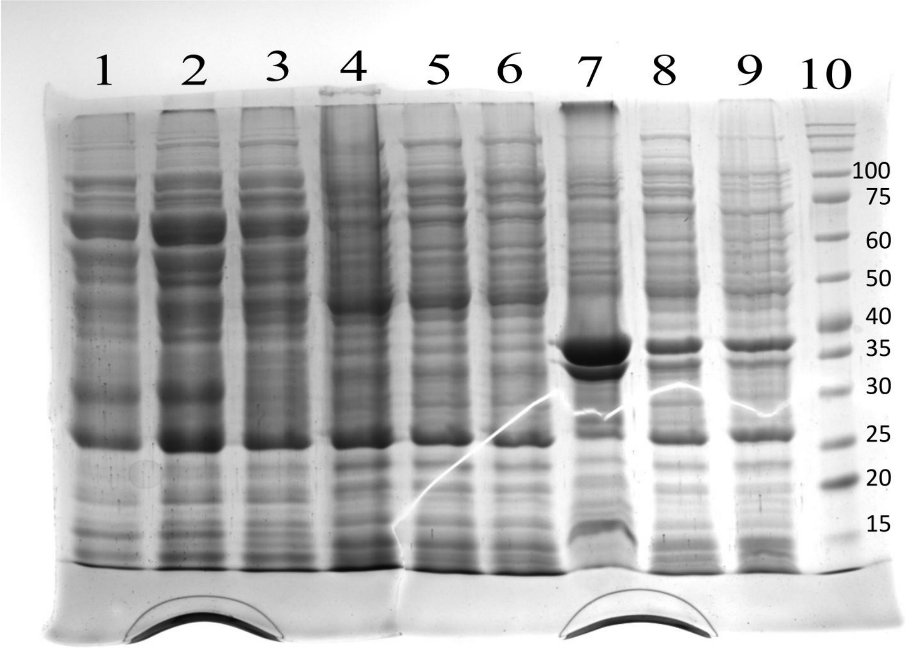

Loading order

1. 600mM imidazole elution
2. 300mM imidazole elution
3. 100mM imidazole elution
4. 20mM imidazole wash
5. Flow through
6. Supernatant
7. Pellet
8. Induced cell lysate
9. Uninduced cell lysate
10. Marker

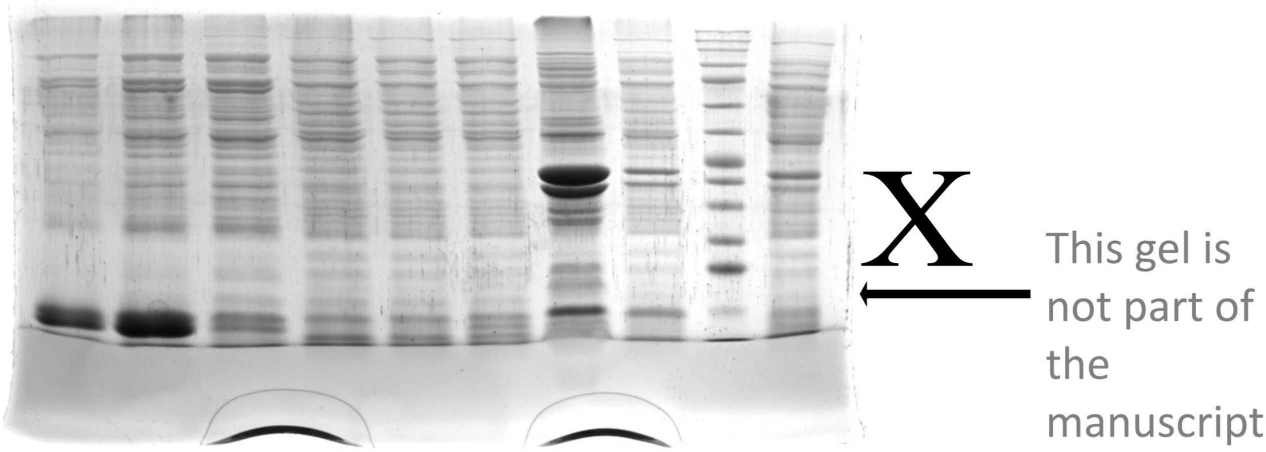

X

This gel is not part of the manuscript

S2c Fig. Periplasmic expression of A32L cloned in pET22b vector

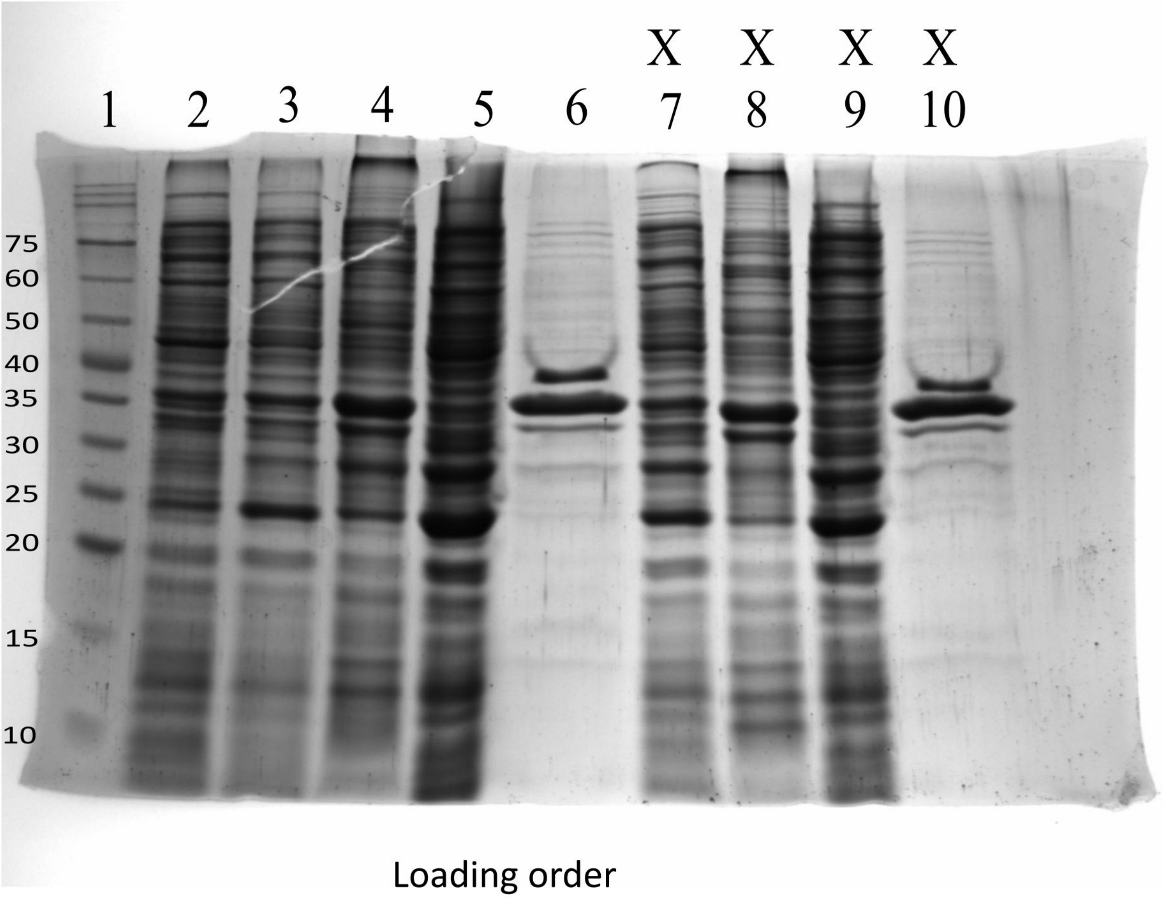

Loading order

1. marker
2. uninduced cells
3. induced cells
4. insoluble pellet
5. cytoplasmic fraction
6. periplasmic fraction
7. induced cells
8. Insoluble pellet
9. cytoplasmic fraction
10. periplasmic fraction
11. empty

Colony 1 (lanes 2–6)

Colony 2 Not shown in manuscript (lanes 7–10)

X- not included in main figure

S2d Fig. Periplasm extraction from *E. coli* cells

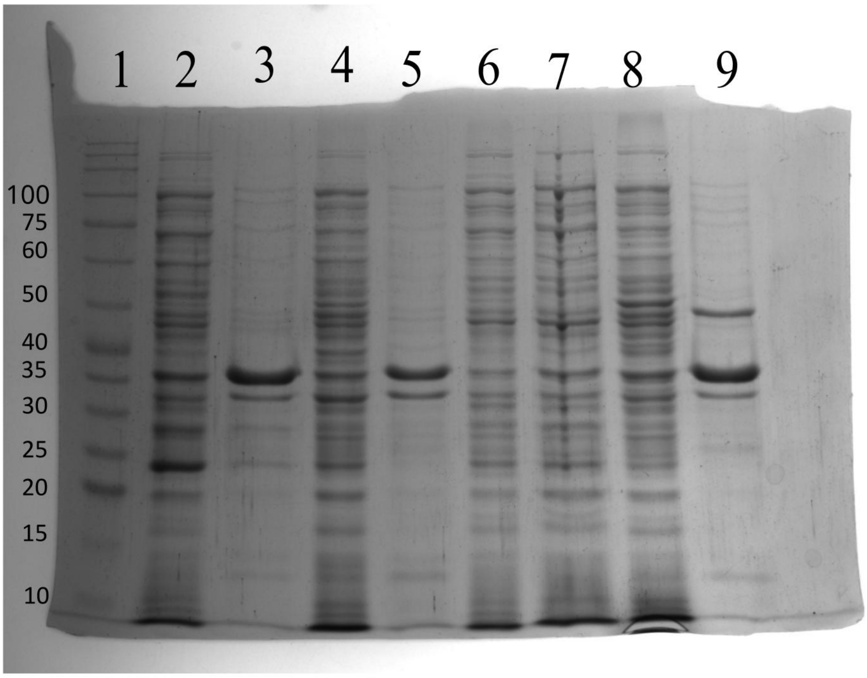

Loading order

1. Marker
2. Induced cells transformed with pET22b-A32
3. Periplasm- induced pET22b-A32

4. Uninduced cells transformed with pET22b-A32
5. Periplasm- uninduced pET22b-A32

6. Induced cells transformed with pET22b
7. Periplasm- induced pET22b

8. Uninduced cells transformed with pET22b
9. Periplasm- uninduced pET22b

S3a Fig. Expression of A32 in recombinant baculovirus-infected sf9 cells.
Cytopathic effects of infecting sf9 cells with recombinant A32 -baculovirus.

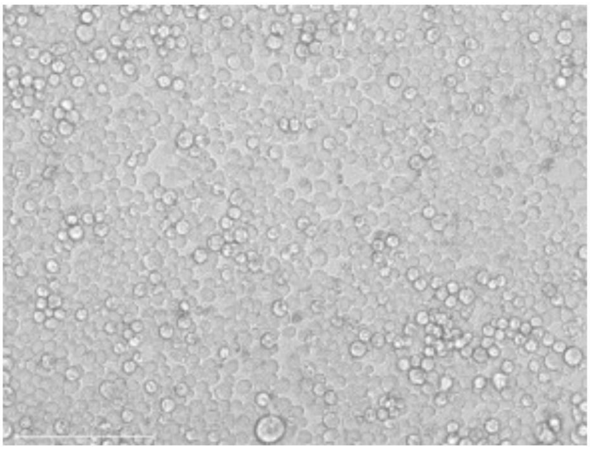

(i)

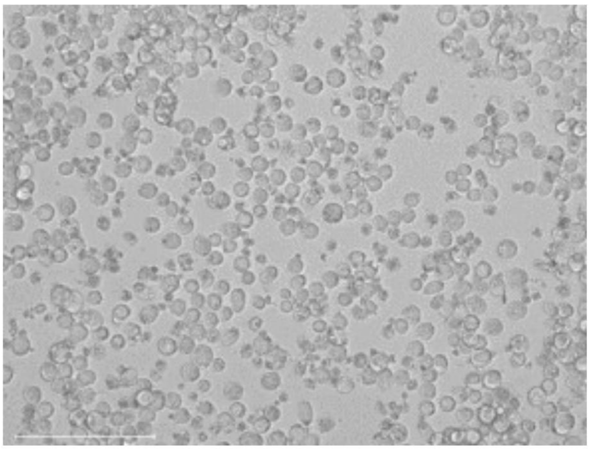

(ii)

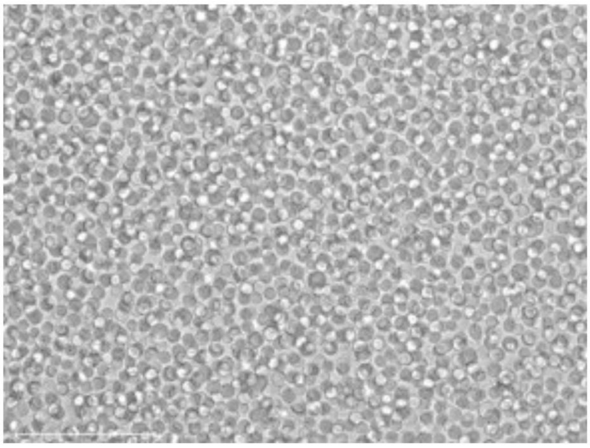

(iii)

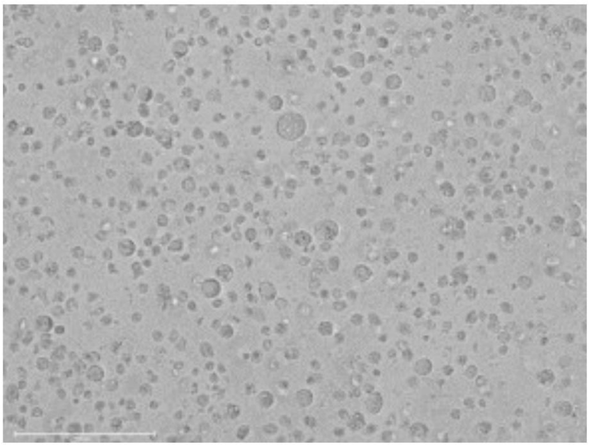

(iv)

(i) uninfected cells 6 dpi
(ii) infected cells 6 dpi
(iii) uninfected cells 8 dpi
(iv) infected cells 8 dpi

Scale bar (white): 150 µM
dpi: days post infection

S3b Fig. SDS-PAGE analysis of sf9 cell lysate infected with baculoviruses

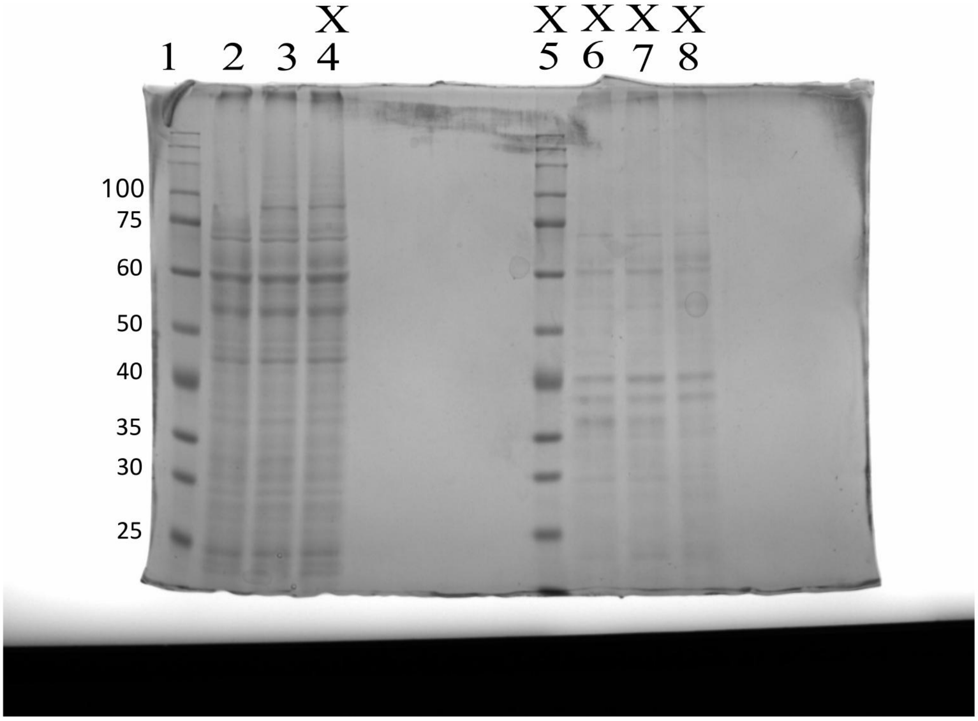

Loading order

1. Marker
2. Wild-type baculovirus infected cell lysate
3. A32-baculovirus infected cell lysate- replicate 1
4. A32-baculovirus infected cell lysate- replicate 2- not shown in manuscript

5-7. Unrelated protein samples- not part of this manuscript

X- not included in final figure

S4a Fig. Expression and glutathione Sepharose bead binding of A32L cloned in pGEX-6P-1 vector

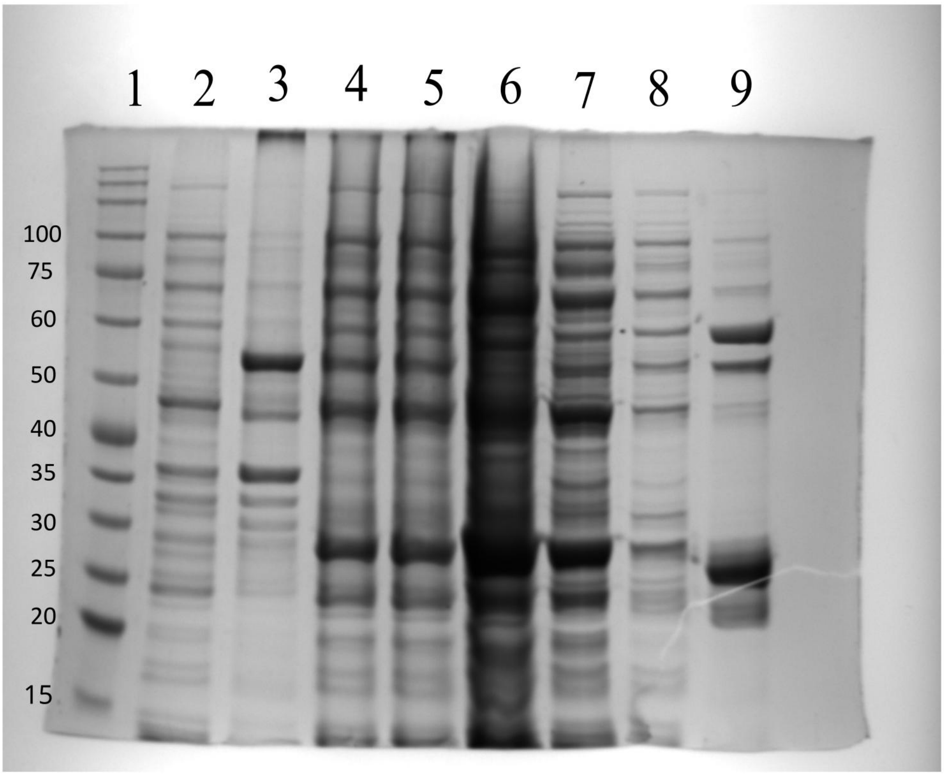

Loading order

1. marker
2. Uninduced cells
3. Pellet
4. Supernatant
5. Flow-through
6. Binding buffer wash 1
7. Binding buffer wash 2
8. Elution 1 with 10mM reduced glutathione
9. Elution 2 with 20mM reduced glutathione

S4b Fig. Western blot with anti-GST antibody

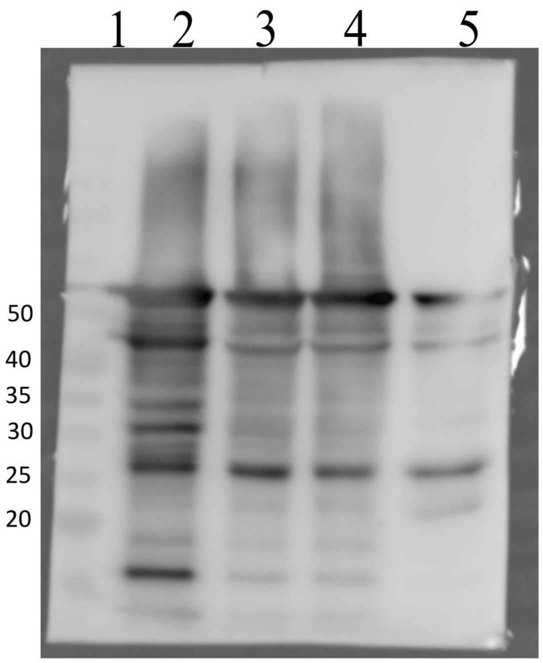

Loading order

1. Marker
2. Pellet
3. Supernatant
4. Flow through
5. Elution

S5a Fig. Cleavage of thioredoxin tag by enterokinase (EK) treatment. $A32_{WT}$ was incubated with enterokinase at 4 °C and 20°C for 14, 17, 36 h

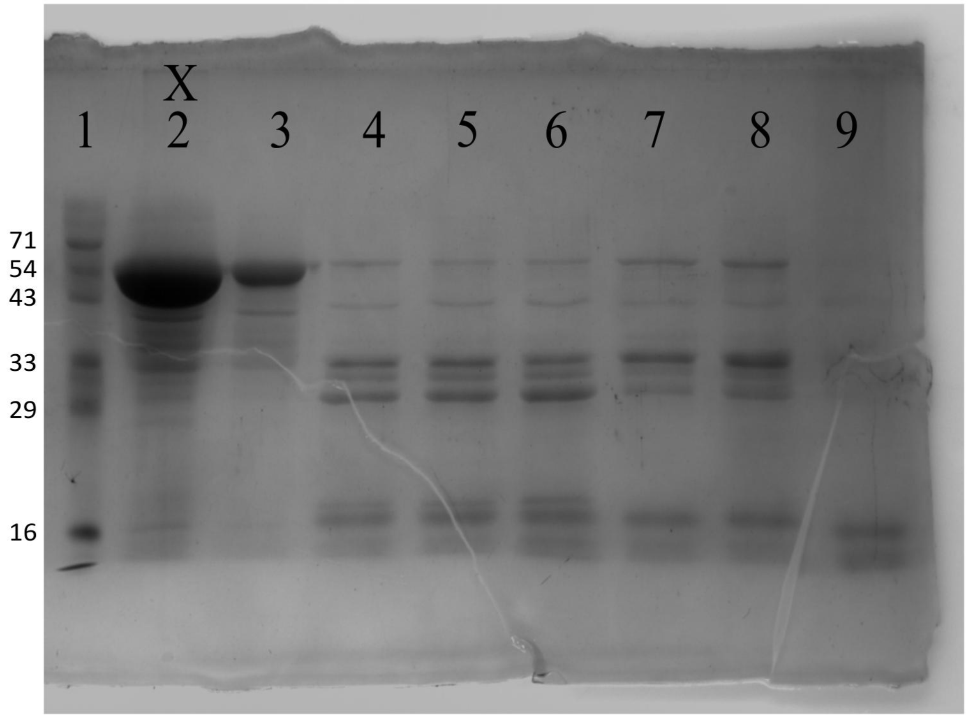

1. Marker
2. Concentrated thioredoxin tagged A32- overloaded
3. Uncut thioredoxin tagged A32- purified and concentrated (1 mg/ml)
4. EK treatment - 4°C, 14hrs
5. EK treatment - 4°C, 17hrs
6. EK treatment - 4°C, 36hrs
7. EK treatment - 20°C, 14hrs
8. EK treatment - 20°C, 17hrs
9. EK treatment - 20°C, 36hrs

X- not included in final figure

S5b Fig. Expression and purification of A32*$_{WT}$ cloned in pET32b-HRV3C plasmid

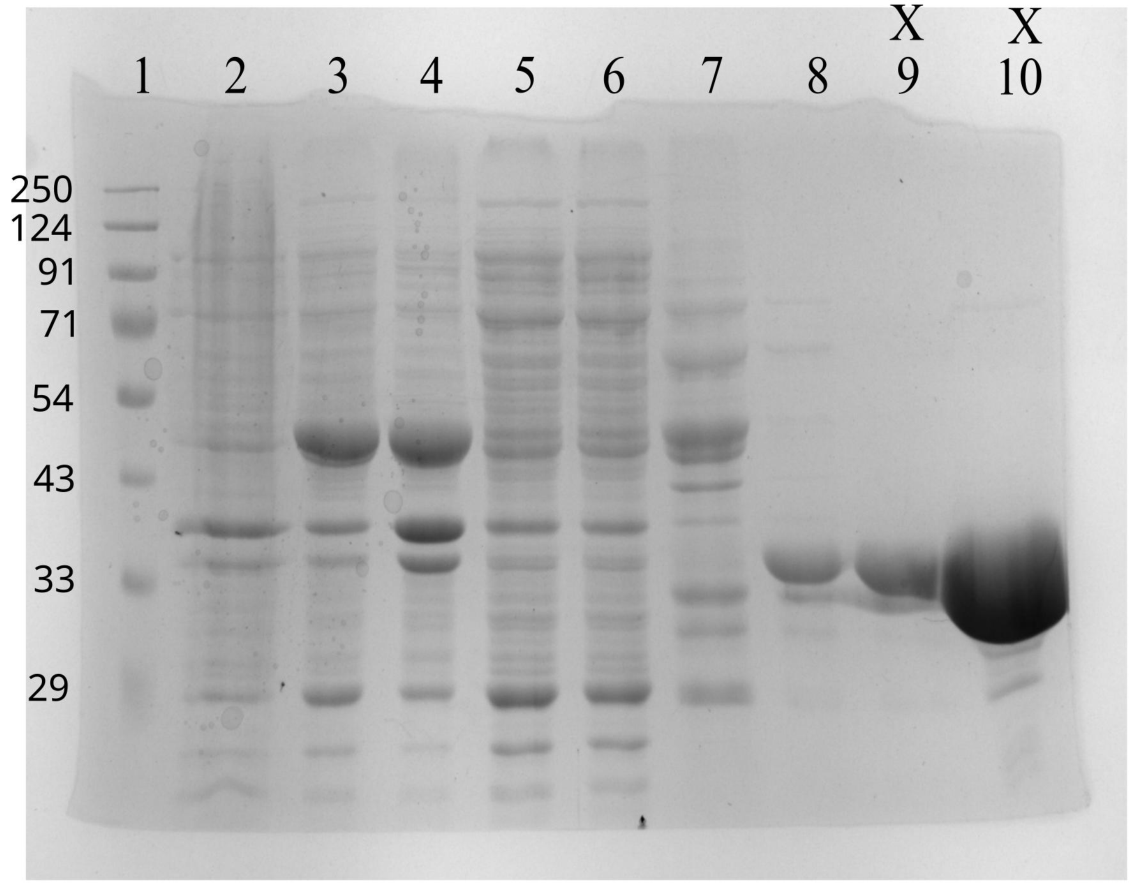

Loading order

1. marker
2. uninduced
3. Induced
4. pellet
5. supernatant
6. flow through
7. partially purified Thioredoxin tagged A32*$_{WT}$ by Ni^{2+}-NTA column chromatography
8. Purified HRV3CP protease
9. Purified HRV3CP protease
10. Purified HRV3CP protease- overloaded

X- not included in final figure

S5c Fig. Cleavage of thioredoxin tag by NT* HRV3CP treatment.

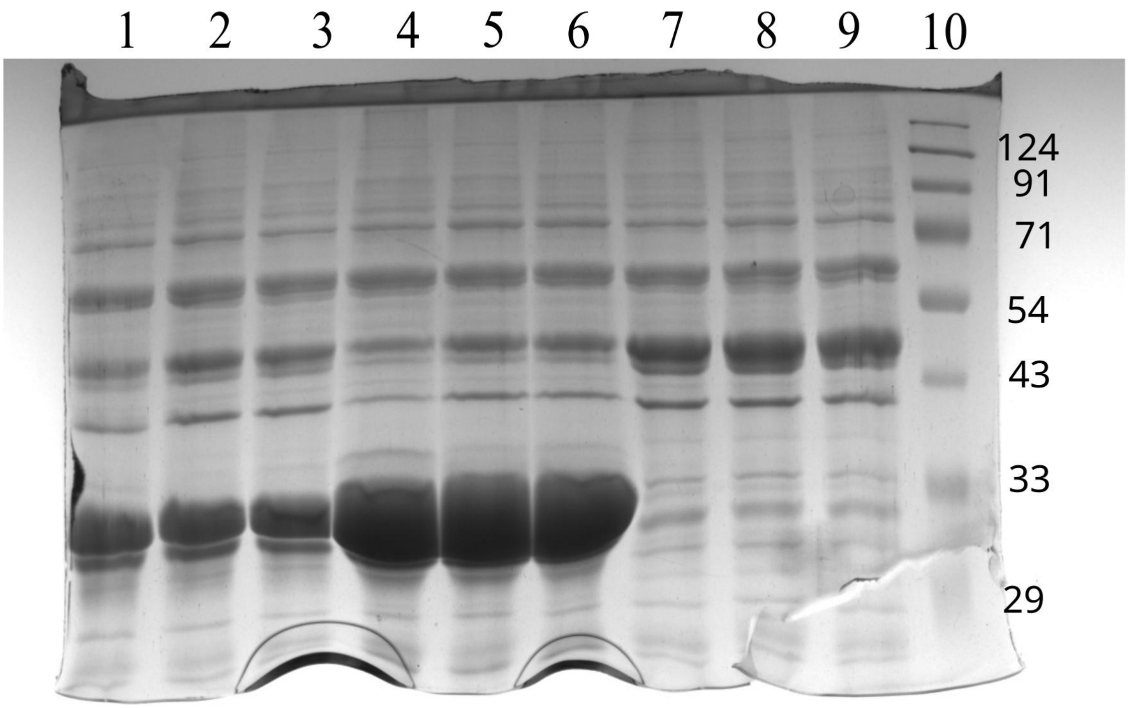

Loading order

1. A32*WT: NT*HRV3CP 1:1 10hr
2. A32*WT: NT*HRV3CP 1:1 2hr
3. A32*WT: NT*HRV3CP 1:1 0hr
4. A32*WT: NT*HRV3CP 1:2 10hr
5. A32*WT: NT*HRV3CP 1:2 2hr
6. A32*WT: NT*HRV3CP 1:2 0hr
7. A32 (protease⁻) 10 hr
8. A32 (protease⁻) 2 hr
9. A32 (protease⁻) 0 hr
10. Marker

S5d Fig. Western blot of A32*$_{WT}$ treated with protease for 10 h using anti-A32 immune sera

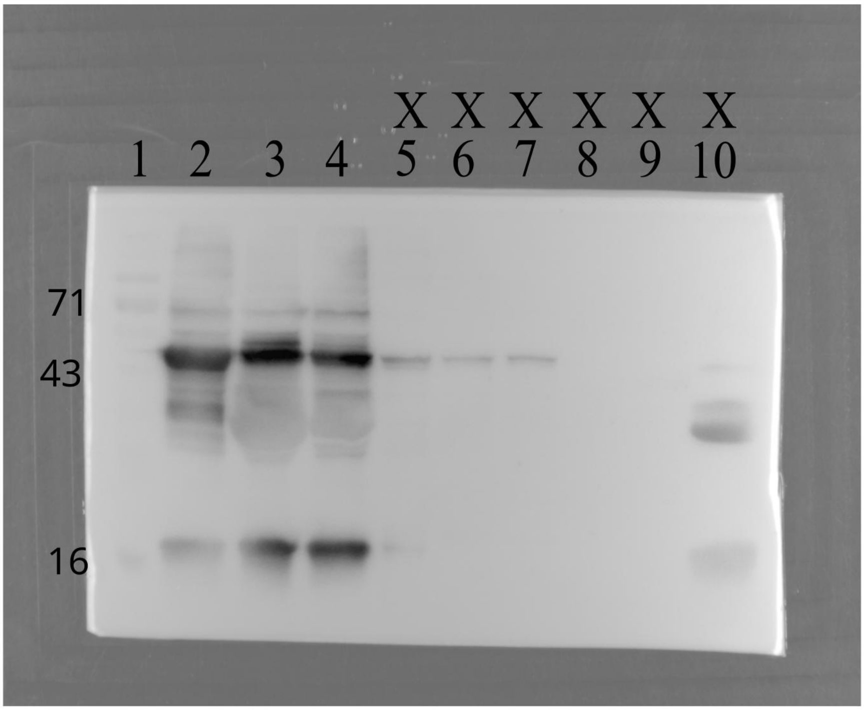

Loading order

1. Marker
2. A32 (no protease) 10 hr
3. HRV: A32 1:1 4°C 10hr
4. HRV: A32 1:2 4°C 10hr

5-7- same as lanes 2-4, less protein volume loaded
8,9- empty
10- unrelated protein sample

X- not included in final figure

S6a Fig. Autoradiographs of steady-state kinetics in the absence or presence of DNA with 6µM A32$_{WT}$

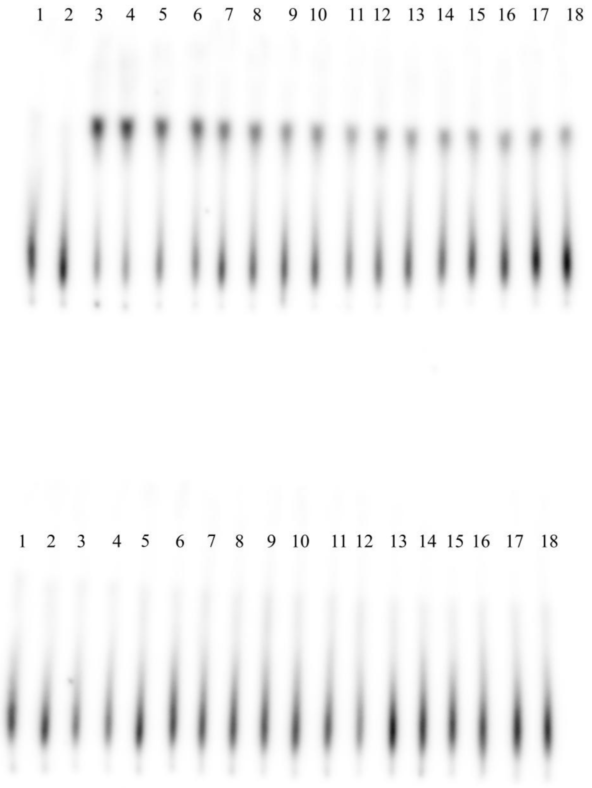

Upper panel- presence of DNA
Lower panel- absence of DNA

1 and 2- A32$_{WT}$ + no ATP
3 and 4- A32$_{WT}$ + 0.1mM ATP
5 and 6- A32$_{WT}$ + 0.2mM ATP
7 and 8- A32$_{WT}$ + 0.4mM ATP
9 and 10- A32$_{WT}$ + 0.6mM ATP
11 and 12- A32$_{WT}$ + 0.8mM ATP
13 and 14- A32$_{WT}$ + 1mM ATP
15 and 16- A32$_{WT}$ + 2mM ATP
17 and18- A32$_{WT}$ + 3mM ATP

S6b Fig. Autoradiographs of steady-state kinetics in the absence or presence of DNA with 6µM Walker A motif mutant $A32_{K31A}$

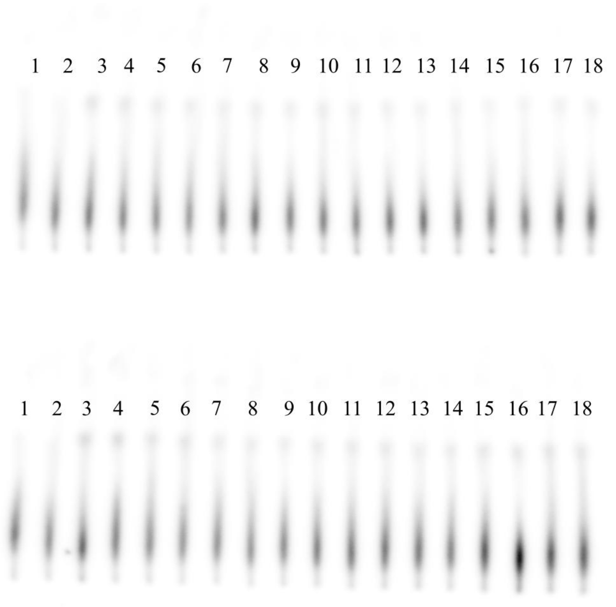

Upper panel- presence of DNA
Lower panel- absence of DNA

1 and 2- $A32_{K31A}$ + no ATP
3 and 4- $A32_{K31A}$ + 0.1mM ATP
5 and 6- $A32_{K31A}$ + 0.2mM ATP
7 and 8- $A32_{K31A}$ + 0.4mM ATP
9 and 10- $A32_{K31A}$ + 0.6mM ATP
11 and 12- $A32_{K31A}$ + 0.8mM ATP
13 and 14- $A32_{K31A}$ + 1mM ATP
15 and 16- $A32_{K31A}$ + 2mM ATP
17 and18- $A32_{K31A}$ + 3mM ATP

S6c Fig. Autoradiographs of steady-state kinetics in the absence or presence of DNA with 6µM coiled coil motif mutant A32$_{L234K_Q237A}$

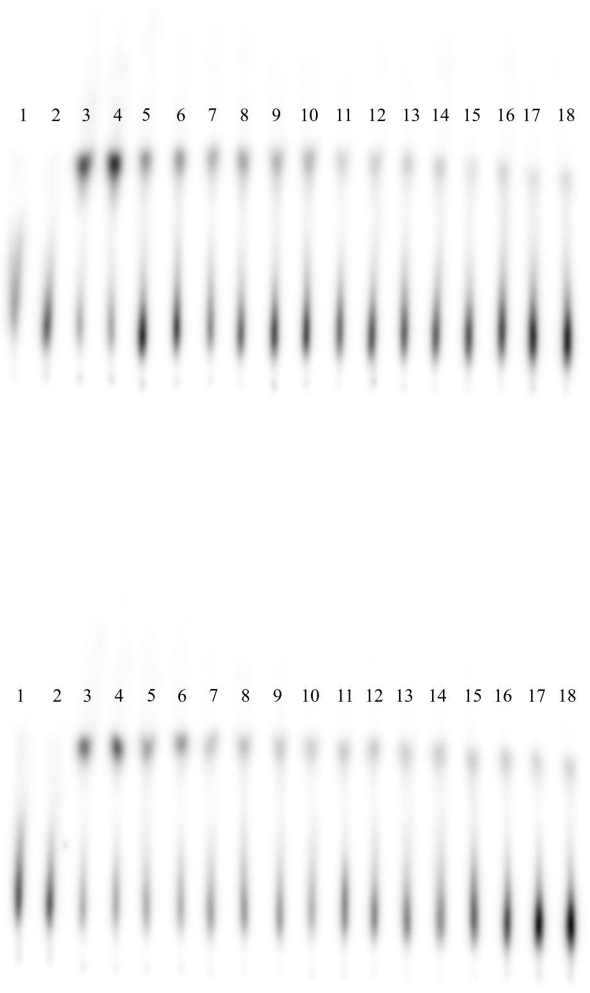

Upper panel- presence of DNA
Lower panel- absence of DNA

1 and 2- A32$_{L234K_Q237A}$ + no ATP
3 and 4- A32$_{L234K_Q237A}$ + 0.1mM ATP
5 and 6- A32$_{L234K_Q237A}$ + 0.2mM ATP
7 and 8- A32$_{L234K_Q237A}$ + 0.4mM ATP
9 and 10- A32$_{L234K_Q237A}$ + 0.6mM ATP
11 and 12- A32$_{L234K_Q237A}$ + 0.8mM ATP
13 and 14- A32$_{L234K_Q237A}$ + 1mM ATP
15 and 16- A32$_{L234K_Q237A}$ + 2mM ATP
17 and18- A32$_{L234K_Q237A}$ + 3mM ATP
